# Exosomal transmission of viruses, a two-edged biological sword

**DOI:** 10.1186/s12964-022-01037-5

**Published:** 2023-01-23

**Authors:** Narges Mardi, Sanya Haiaty, Reza Rahbarghazi, Halimeh Mobarak, Morteza Milani, Amir Zarebkohan, Mohammad Nouri

**Affiliations:** 1 Department of Medical Biotechnology, Faculty of Advanced Medical Sciences, Tabriz University of Medical Sciences, Tabriz, Iran; 2 Infectious and Tropical Diseases Research Center, Tabriz University of Medical Sciences, Tabriz, Iran; 3 Stem Cell Research Center, Tabriz University of Medical Sciences, Imam Reza St., Golgasht St., Tabriz, Iran; 4 Department of Applied Cell Sciences, Faculty of Advanced Medical Sciences, Tabriz University of Medical Sciences, Tabriz, Iran; 5 Department of Medical Nanotechnology, Faculty of Advanced Medical Sciences, Tabriz University of Medical Sciences, Tabriz, Iran

**Keywords:** Viruses, Exosomes, Shared signaling pathways, Transmission, Infection

## Abstract

As a common belief, most viruses can egress from the host cells as single particles and transmit to uninfected cells. Emerging data have revealed *en bloc* viral transmission as lipid bilayer-cloaked particles via extracellular vesicles especially exosomes (Exo). The supporting membrane can be originated from multivesicular bodies during intra-luminal vesicle formation and autophagic response. Exo are nano-sized particles, ranging from 40–200 nm, with the ability to harbor several types of signaling molecules from donor to acceptor cells in a paracrine manner, resulting in the modulation of specific signaling reactions in target cells. The phenomenon of Exo biogenesis consists of multiple and complex biological steps with the participation of diverse constituents and molecular pathways. Due to similarities between Exo biogenesis and virus replication and the existence of shared pathways, it is thought that viruses can hijack the Exo biogenesis machinery to spread and evade immune cells. To this end, Exo can transmit complete virions (as single units or aggregates), separate viral components, and naked genetic materials. The current review article aims to scrutinize challenges and opportunities related to the exosomal delivery of viruses in terms of viral infections and public health.

**Video Abstract**

**Supplementary Information:**

The online version contains supplementary material available at 10.1186/s12964-022-01037-5.

## Introduction

Each cell can release different types of extracellular vesicles (EV) into the extracellular matrix (ECM) under physiological and pathological conditions [1]. Among EVs, exosomes (Exo) have been considered significant biological tools in cell-to-cell paracrine activity [1]. The existence of diverse biological factors inside the Exo highlights their critical role of them in the transport of signaling biomolecules [2]. Various lipids, nucleic acids (miRNAs, lncRNA and circRNA, mitochondrial DNA, and single and double-strand DNA), and growth factors can be found in the lumen of Exo [2]. Because Exo biogenesis and abscission are tightly regulated by varied effectors from different pathways [3], it is logical to hypothesize that Exo production can affect other cellular activities or vice versa [4]. In support of this claim, molecular investigations have shown similarities between the Exo biogenesis and assembly and egress system of several viruses [5]. During the last decades, this phenomenon has led to the rise of the Trojan Exo hypothesis demonstrating that different viruses can hijack the Exo trafficking system to spread between the cells without the direct interaction between viral ligands and host cell receptors [6]. The identification and presence of viral particles inside the releasing Exo supports the integration of the viral and Exo assembly systems [7]. Whether and how viruses can hijack the Exo trafficking system is the subject of debate. Here, we aimed to highlight the possible interaction between viral assembly machinery with the Exo trafficking system. Beidses, different mechanisms viruses can use for propagation and infection via Exo will be discussed.

## Exosome biogenesis and abscission

**Fig. 1:**
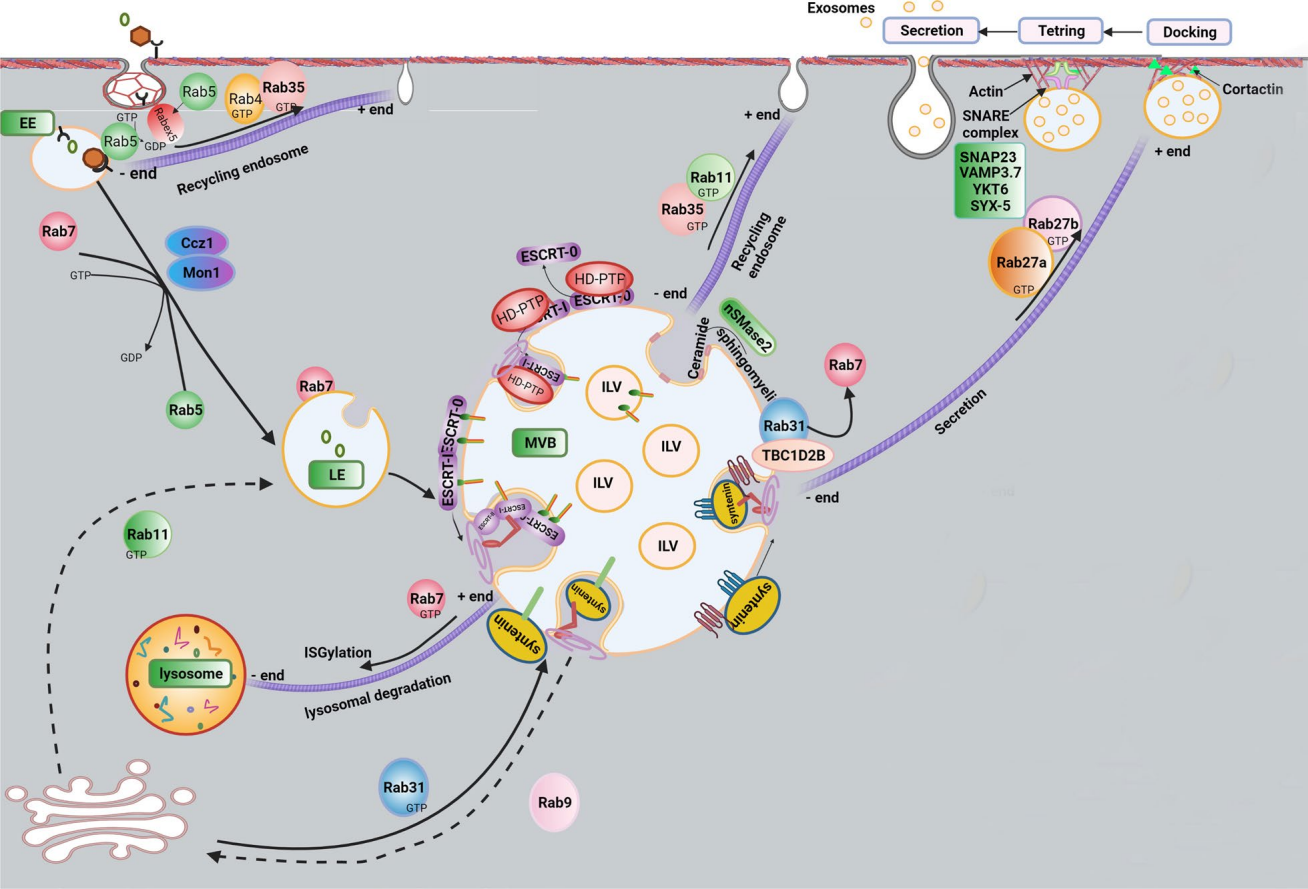
The generation of Exo is regulated by multiple intracellular pathways. Cargoes are sequestrated into early endosomes which are originated from endocytosis (Rab4, Rab5, Rab7, and Rab35) or Trans-Golgi network (Rab11). Exo fusion and recycling back pathways are regulated by the activity of Rab proteins (Rab 27a and Rab27b), SNARE complex (SYX-5, YKT6, VAMP3.7, SNAP23). The formation of ILVs inside the MVBs is mediated by inward invagination endosomal membrane via multiple pathways: ESCRT-dependent (ESCRT-0, I, II, II and syndecan-syntenin-ALIX axis), and ESCRT-independent pathways (ceramide-enriched microdomains and tetraspanin-enriched microdomains). Multiple pathways can distinguish the orientation of MVBs toward lysosomal depredation or fusion with the plasma membrane. ISGylation process can induce lysosomal degradation. The lysosomal degradation pathway is regulated by interaction Rab7 with Dynein and induction mobility toward microtubule minus ends. To fuse the MVBs with the membrane, various Rab-GTPases control the transport of MVBs on microtubules. Rab27 stabilizes the rearrangement of the actin cytoskeleton by improving the attachment of Cortactin, leading to MVBs docking. Docking, tethering, and releasing are three steps. The activity of SNAREs mediates the fusion of the MVB membrane with the plasma membrane

**Fig. 2:**
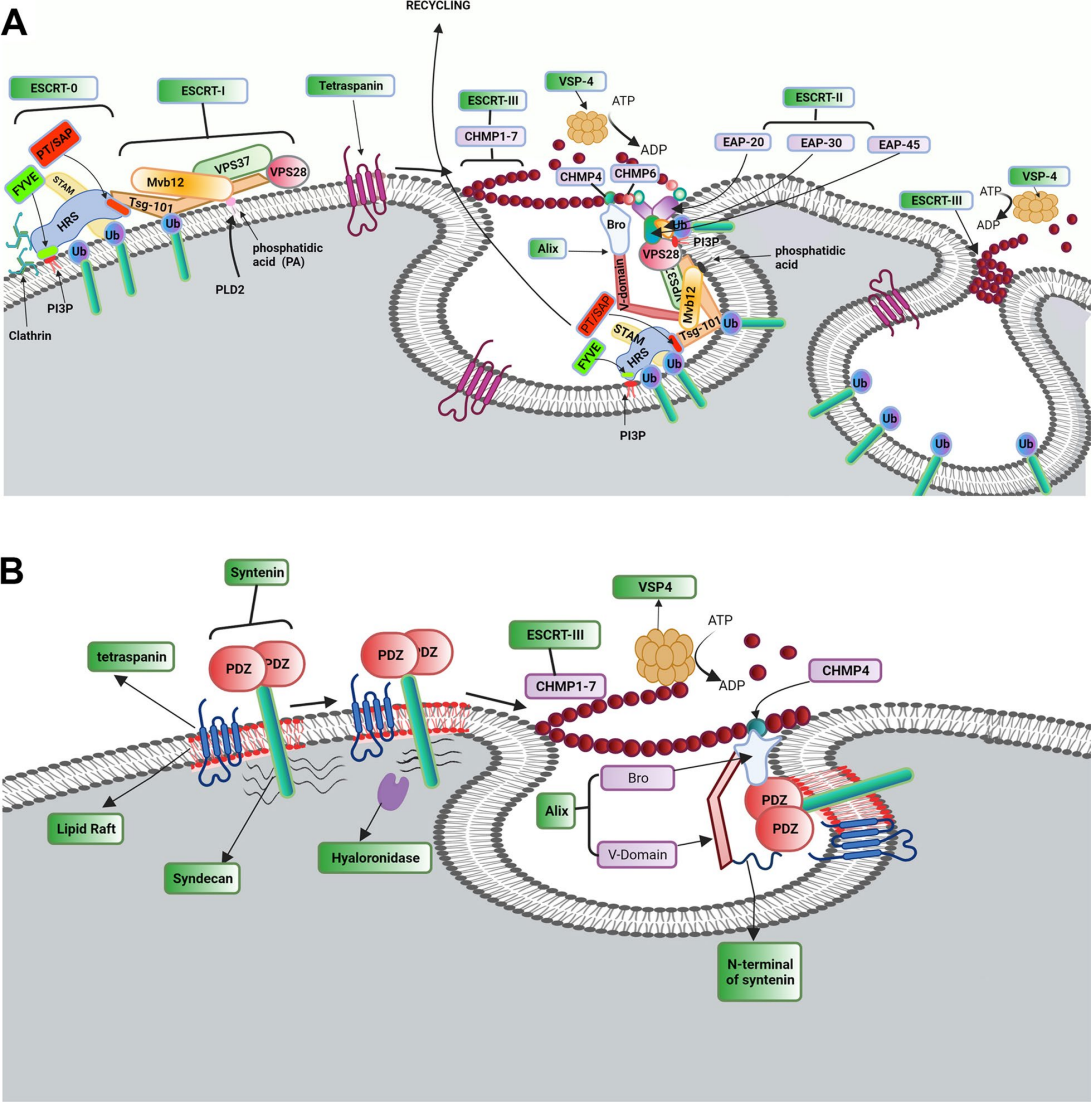
Exo biogenesis via conventional ESCRT-dependent pathway (**A**). Conventional ESCRT-dependent strategy can lead sorting of four ubiquitinated cargos into ILVs. ESCRT machinery is composed of ESCRT-0, -I, -II, and -III. The recruitment of ESCRT-0 occurs on the cytoplasmic side of the endosomal membrane. ESCRT-0 complex consists of HRS and STAM subunits to recognize the ubiquitinated cargoes. The recruitment of ESCRT-0 is triggered via the interaction of the ESCRT-0 HRS subunit with PIP3 on the endosomal membrane. The interaction of ESCRT-0 HRS with clathrin proteins enhances the clustering of ESCRT-0 to endosomal membrane microdomains. To select exosomal cargo, ESCRT-0 provides a platform for the attachment of ESCRT-I. ESCRT-I consists of four subunits: TSG-101, Mvb12, VPS37, and VPS28. Interaction between ESCRT-0 HRS subunit with ESCRT-1 TSG-101 subunit leads to physical attachment of ESCRT-0 and ESCRT-1 on endosome membrane. ESCRT-0 and -I proteins provide a binding site for of ESRT-II complex (EAP45, EAP30, and two EAP20). The physical connection is done via the ESCRT-I VPS28 subunit and ESCRT-II EAP45 subunit. Interaction of ESCRT-I with ESCRT-II can induce invagination of the endosomal membrane. The complex of ESCRT-0, -I, and -II can induce the assembly and polymerization of ESCRT-III subunits (CHMP-1, -2, -3, -4, -5, -6, and -7). Interaction of EAP20 of ESCRT-II with CHMP6 of ESCRT-III promotes the recruitment of ESCRT-III subunits. The activation of ESCRT-III promotes a chain around the neck of intraluminal vesicles. Endosomal membrane curvature is stimulated by the interaction of Alix with Mvb12 and CHMP4 subunits of ESCRT-I and ESCRT-III, respectively. Upon ILV scission, Vps4-ATPase induces ESCRT-II subunits disassociation. Role of Syndecan-Syntenin-Alix in Exo biogenesis (**B**). Syndecan-Syntenin-Alix stimulates Alix-ESCRT-mediated sorting in Exo. Heparanase induces Syndecans clustering and facilitates its attachment to adaptor protein Syntenin via PDZ domains. Syntenin N-terminus connects to Alix via the direct interaction with V-domain. In the end, VSP4 is recruited by the ESCRT-III complex and leads a session of ILVs into MVB

Exo are originated from intracellular vesicles namely intraluminal vesicles (ILVs) (Fig. 1). The concept of ILVs implies the invagination of the endosomal membrane into the lumen that promotes the formation of numerous nano-sized vesicles [8, 9]. Further fusion of the endosomal compartments with plasma membrane triggers the release of ILVs into the ECM where they are, hereafter, known as Exo with an average size of 30–150 nm [8–10]. The physiological significance of Exo is related to the transfer of different biomolecules between the cells during different contexts, suggesting that Exo act as natural bio-shuttles inside the body [11]. The procedure of Exo biogenesis is summarized in three steps as follows; formation, transfer, and release (abscission) [12]. The formation of Exo ancestors is triggered by inward budding and de novo formation of early endosomes through the endosomal pathway [13, 14]. As an alternative pathway, early endosomes are derived from the Trans-Golgi network and can fuse with the pre-existing early endosomes [13, 14]. In later phases, early endosomes mature into late endosomes. During endosomal maturation, Rab 5 is replaced with Rab 7 via a guanosine exchange factor namely Mon1–Ccz1 complex [15]. It was suggested that Rab 7 is a regular protein in several processes related to endosomal maturation, degradation, and secretion [15]. Inside late endosomes, the membrane is actively invaginated into the luminal surface to constitute numerous ILVs resulting in the formation of multi-vesicular appearance namely multivesicular bodies (MVBs) [16]. There are multiple MVB biogenesis mechanisms as follows; ESCRT-dependent and ESCRT-independent mechanisms [17]. It was suggested that the ESCRT-dependent pathway is classified into canonical and non-canonical signaling cascades [18]. In the canonical ESCRT-dependent pathway, ESCRT-0, its HRS domain with tyrosine kinase activity is recruited to the endosomal membrane via phosphatidylinositol-3-Phosphate binding (Fig. 2A) [19]. This complex can recognize ubiquitinated proteins and sequestrate into the luminal surface of vesicles [19]. In the latter steps, ESCRT-0, as a member of the sorting machinery system, is recalled followed by the addition of ESCRT-I, -II, and –III [20]. Among them, the collaboration of the collection of ESCRT-0, -I, and II facilitates the invagination of endosomal membranes and enhances the sorting efficiency of ubiquitinated proteins within the buds [20]. Upon the recruitment of ESCRT-III, ESCRT-III components are dynamically assembled to promote ILV formation and abscission from the MVB membrane [20]. After the completion of this step, ESCRT-III is disassociated via the activation of AAA-ATPase Vps4 [21].

The non-canonical ESCRT-dependent mechanisms can participate in the formation of ILVs in yeasts, and some species of mammals [22]. For this purpose, the Histidine domain protein tyrosine phosphatase (HD-PTP) acts as a scaffold protein and binds the ESCRT complex in the absence of ESCRT-II [18]. It has been shown that there are other non-canonical ESCRT-dependent mechanisms activated by the syndecan-syntenin-ALIX axis (Fig. 2B) [23]. Of note, syndecan, as a transmembrane protein, interacts with syntenin to recall ALIX [23]. The final complex is not involved in the ubiquitination of proteins. To be specific, in the syndecan-syntenin-ALIX axis, both ESCRT-III and VPS4 actively regulate membrane budding and cargo sorting [24]. A plethora of documents has shown that ESCRT-independent mechanisms are also involved in ILV biogenesis [25]. Noteworthy, sphingomyelin located on the endosomal membrane is further cleaved to ceramide and phosphorylcholine by the activity of neutral sphingomyelinase 2 (nSMase 2) [26]. The collaboration of Ceramide with the ESCRT-independent pathway forms lipid raft microdomains at the surface of the endosomal membrane [27]. As a correlate, this complex induces negative curvature of the ceramide-enriched membrane and enforces ILV into the MVB lumen [27]. Molecular investigations have revealed that other effectors such as Rabs can also orchestrate the process of ILV formation via ESCRT-independent mechanisms [28]. For instance, RAB31 controls ceramide-dependent ILV budding procedure [28]. Like RAB31, some tetraspanin subsets such as CD63 play an important role in the ESCRT-independent pathway of ILV biogenesis [25]. This protein can stimulate the formation of tetraspanin-enriched microdomains, participating in ILVs biogenesis [29]. Based on the numerous findings, three destinations have been proposed for second endosomes inside each cell as follows; abscission, enzymatic digestion, and recycling [25]. First, these particles are directly guided to lysosomes where they undergo enzymatic degradation [25]. In the latter scenario, MVBs can fuse with the plasma membrane and shed their contents (hereafter known as Exo) into the neighboring ECM [8, 30]. Some fraction of released Exo can return to the host cells through the recycling mechanisms. This action is done by back-fusion with the plasma membrane and the participation of specific elements related to secretion pathways [31]. To orientate MVBs toward enzymatic digestion or abscission procedure, effectors from the Rab-GTPase family such as Rab7, 11, 35, and 27 are involved [3]. For example, Rab7 mediates MVB trafficking to lysosomal degradation. Rab11, 27, and 35 in collaboration with the SNARE complex control the secretion of ILVs out of the cells [31]. Molecular identification of Exo has shown complex cargo sorting and selective genomic, proteomic, and lipidomic contents [32]. This would occur in the parent cells where all steps correlated with Exo formation, transfer, and release happen. Therefore, it is logical to mention that the metabolic status of host cells contributes to the diversity of Exo cargo type [32]. Based on recent studies, it has been proposed that the final composition of EVs is relatively different from the parental cells [33]. This apparent difference may relate to the engagement of specific cargo sorting machinery with highly regulated activities that increase the density of certain factors inside the ILVs [33]. Noteworthy, some of the sorted biomolecules are common among all Exo like antigen-presentation proteins, adhesion molecules, motility factors, heat shock proteins (HSPs) and chaperones, MVB biogenesis associated factors, tetraspanins, trafficking and membrane fusion proteins, LAMP (lysosome-associated membrane glycoprotein), GTPases and metabolic enzymes [34–36]. Exo can harbor genomic content such as DNA, RNA, coding, and non-coding RNAs. Other genomic components such as mRNA, miRNA, rRNA, tRNA, vault RNA, Y-RNA, and circular RNA have been previously determined inside the Exo [33, 37]. A quantitative genomic analysis has revealed that the nucleotide content of Exo cargo differs, if not completely but in part, from the donor cell profile [38]. Data suggest the participation of specific sequence motifs in the loading of certain RNAs into the Exo lumen. Like RNAs, several types of DNA such as ssDNA, dsDNA, and mtDNA are sorted into the Exo. Of note, the manipulation of host cells to deliver DNA horizontally into the other cells is limited [38]. Variable levels of lipids including Cholesterol, Ceramides, Sphingomyelin, Phosphatidylserine, Gangliosides, and saturated fatty acids exist inside the Exo [39]. A piece of evidence points to the fact that lipid composition can affect Exo stability, trafficking, recognition, and internalization rate [40]. The existence of a significant difference in the lipid content of Exos isolated from plasma suggests that plasma harbors several arrays of Exos that originated from different cell sources. Alternatively, certain molecular pathways are engaged to sort specific lipids into the Exo [41]. Like lipid profile, genomic and proteomic profiles, results showed a mild to moderate increase of carbohydrates inside the Exo compared to the cytosol. For instance, Mannose, complex N-linked glycans, poly-lactosamine, and sialic acid are enriched into the Exo during the synthesis procedure [33, 42]. It is believed that the type and content of carbohydrates can change the exosomal protein content. Among carbohydrates, glycans act as a sorting device of proteins [42]. Concerning sorting activity, recent experiments have shown the crucial role of the ubiquitination process in the direction of different proteins into the Exo.

Of several intracellular mechanisms, the ESCRT accelerates the sorting of ubiquitinated proteins into the ILVs, furthermore, it is involved in the formation of ILVs [37], cytokinesis, and the invagination of some of the viruses. This complex is a modular super complex and consists of four components ESCRT-0, -I, -II, and -III, and accessory complexes Vps4 and Alix. Endosomal sorting is closely associated with the activity of the ESCRT system for the regulation of specific cargoes into ILVs inside MVBs [43, 44].

In the early stages, the factor ESRT0 initiates the formation of MVBs [45]. This factor is a heterodimer protein with two subunits, HRS (HGF-regulated tyrosine kinase substrate) and STAM (signal transducing adaptor molecule) [46]. It is believed that the promotion of HRS correlates with the formation of ILVs [47]. To this end, HRS attaches to phosphatidylinositol-3- phosphate (PI3P) on the endosomal membrane by the FYVE zinc finger domain, and the carboxy-terminal clathrin box of HRS contributes to HRS clustering into clathrin endosomal microdomains [48, 49]. Following the clathrin removal by V-ATPase HSC70, the formation of ILVs continues [32, 50]. The complex of HRS and STAM can bind to the clusters of ubiquitinated proteins by ubiquitin-binding domains, and ubiquitinated cargoes are directed to efficient sorting and degradation [51]. In later steps, HRS of ESCRT-0 binds to the amine terminal of the TSG101 subunit of ESCRT-I via P (T/S) A P motif in the carboxy-terminal [52–56].

ESCRT-I is a soluble hetero-tetramer protein complex consisting of four subunits: TSG101, Vps28, Vps37, and Mvb12 [57–61]. Protein–protein interaction between HRS of ESCRT-0 and TGS101 of ESCRT-I results in the recruitment of ESCRT-I from the cytoplasm to endosomal microdomains [60]. Furthermore, a recently conducted study has shown that produced phosphatidic acid by phospholipase-D2 interacts with Mvb12, an ESCRT-I subunit. As a correlate, phospholipase D2 activity supports the recruitment of ESCRT-I on the endosomal membrane and in the end, directs ESCRT-dependent endosomal maturation [62]. It has been proposed that ESCRT-I is a rod-shaped complex and is a bridge adapter to connect TSG101 with HRS while VPS28 simultaneously is attached to the specific residue of EAP45 belonging to the ESCRT-II complex [63–65]. Besides, TSG 101 can directly interact with ubiquitinated proteins through the UEV domain [66]. The collaboration of ESCRT-I with -II, triggers the invagination of the endosomal membrane into the lumen [67, 68].

Like ESRT-I, ESCRT-II plays an important role in membrane budding, MVB biogenesis, bridging ESCRT complexes to each other [69], and probably communicating between MVBs and microtubules via RILP, Rab7, and dynein [70–72]. ESCRT-II is a hetero-tetramer complex with a Y-shaped structure, composed of EP45, EP30 attaching section, and two EP20 as branch structures. The complex v binds to PI3P via the Glue domain of the EAP45 subunit preferentially, ubiquitinated cargos, and VPS28 of ESCRT-I [54, 56, 57]. In addition, ESCRT-II interacts with two ESCRT-III complexes via engaging two EAP20 subunits [73, 74].

Unlike ESCRT-0, -I, and -II, the ESCRT-III complex is vulnerable to cytosol niche due to a lack of pre-assembled structure [69, 75]. The formation of the ESCRT-0, -I, -II complex recalls the ESCRT-III components and stimulates the transient polymerization of helical filaments on the endosomal membrane [69, 75]. Molecular investigation revealed that the interaction between C-N units initiates ESCRT assembly. It is thought that ESCRT-III is composed of seven different subsets including CHMP 1, 2, 3, 4,5,6, and 7. Among them, the factor CHMP6 recalls the EAP20 in the structure of ESCRT-II. By the progression of these steps, the ESCRT-III complex facilities the budding vesicles into the endosome lumen via collaboration with a specific complex consisting of two hexameric or heptameric rings namely the AAA-ATPase Vps4 complex. The ATPase activity of Vps4 is regulated by the stimulation of an adaptor protein Vta1[69, 76]. The recruitment of other effectors such as Alix and TSG101 support molecular bridges for the attachment of ESCRT-I and -III [77, 78]. In addition, the formation of syndecan-syntenin-Alix can provide a niche to attach this complex to the ESCRT machinery system involved in the cargo sorting of protein inside the ILVs independent of ubiquitination [79, 80]. During the ILV formation, the importance of other components such as lipids has been proved. Ceramides form specific membrane microdomains at the surface of the endosomal membrane and induce negative curvature of the ceramide-enriched membrane. In addition to ILV formation, Ceramides are subsequently converted to sphingosine 1 phosphate and sphingosine via metabolic reactions, acting as lipid mediators [32, 81, 82]. For example, sphingosine 1 phosphate binds to the sphingosine 1 phosphate receptor coupled with inhibitory G protein involved in the cargo sorting into ILVs and the exosomal maturation inside the MVBs [32, 81]. Cholesterol-rich lipid rafts act as a platform for signaling coordination through receptors such as GPI-binding proteins, tyrosine kinases of the Src family, and Ca^2+^ channels [32, 83, 84].

Previous studies support the role of Tetraspanins respectively CD63, CD9, and CD81 in the ESCRT-independent pathway of Exo biogenesis [85–87]. These proteins can modulate cell adhesion, signaling, invasion, membrane fusion, trafficking, and membrane abscission [88, 89]. Tetraspanins with conserved transmembrane structures are organized in the membrane microdomains-rich cholesterol; Tetraspanins, as adaptors for other proteins, through interaction with Tetraspanins and other transmembrane and cytosolic proteins are involved in the formation of specialized membrane platforms called Tetraspanin membrane microdomains (TEM) [90, 91]. Of note, CD63, as a transmembrane protein can interact with Syntenin to form the CD63-Syntenin-ALIX complex which is involved in the MVB biogenesis [92]. Other Tetraspanin members such as CD81 and CD9 can attach to cytoskeletal actin and endosomal membrane microdomains via G proteins [88, 93]. However, a direct association between the CD81 and Rac GTPase in TEM is proved [94, 95]. On the other side, the interaction of CD9 with exosomal integrin is involved in targeting Exo to the specific tissue.

**Fig. 3:**
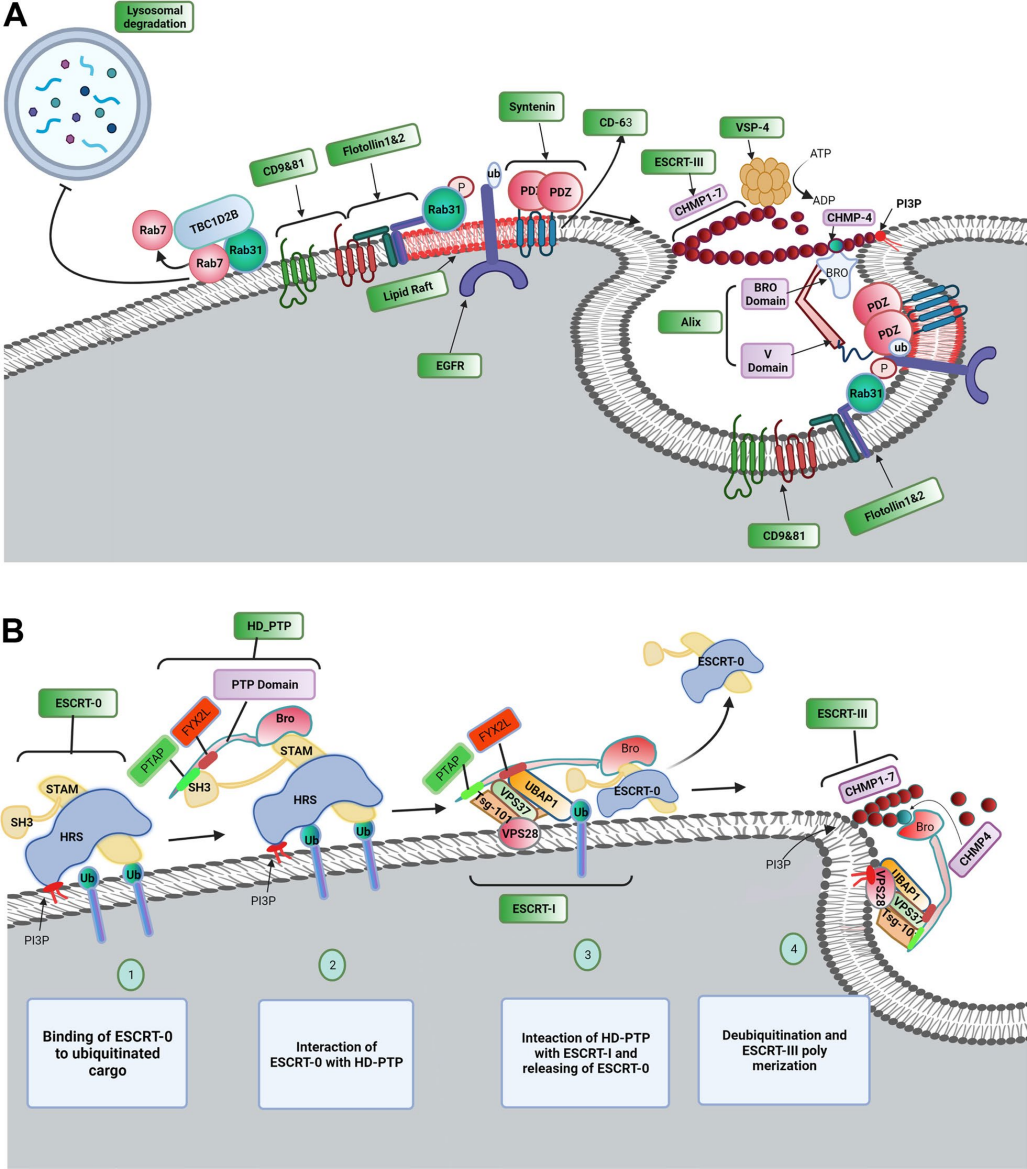
RAB31 inhibits the lysosomal degradation of MVBs (**A**). The existence of a tyrosine kinase receptor namely EGFR on the late endosomal surface activates Rab31 via tyrosine phosphorylation. The phosphorylated Rab31 interacts with flotillin proteins. Rab31-flotillin acts as a scaffold heterodimer protein and forms a budding platform for sorting EGFR and other proteins into MVB lumen in collaboration with lipid rafts. On the other hand, Rab31 recruits TBC1D2B, and the Rab31-TBC1D2B complex inactivates Rab7, preventing the lysosomal degradation of MVBs. Besides the Syntenin-Alix-ESCRT-III, Rab31-flotillin complex acts as parallel sorting machinery that drives different cargoes such as CD63, CD81, and CD9 into exosomal pathways. CD63 is a membrane protein that can interact with PDZ domains of Syntenin. Interaction N-terminal of Syntenin with the V-domain of Alix induces the recruitment of ESCRT-III by the Bro domain of Alix and the formation of ILVs. HD-PTP acts as a scaffold for the binding of ESCRT subsets during the trafficking of ubiquitinated cargo into the MVB lumen (**B**). HRS and STAM are subunits of ESCRT-0 and can bind to the ubiquitinated cargos on the endosomal membrane. STAM is composed of STAM (GAT) and SH3 domains. STAM domain interacts with the Bro1 domain of HD-PTP and the SH3 domain is linked to the *PPRPTAPKP* motif in the PTP domain of HD-PTP. ESCRT-0 is dissociated from HD-PTP by the interaction of ESCRT-I TSG-101 with the PTAP motif of the PTP domain. Then, the ESCRT-I UBAP1 subunit can interact with the FYX2L motif in the CC region of the PTP domain. In the later phase, the interaction of ESCRT-III CHMP4 subunit with the Bro domain of HD-PTP enhances the dissociation of ESCRT-0 from HD-PTP, facilitates polymerization of ESCRT-III, and drives the ubiquitinated cargo into the MVB pathway

Upon the formation of ILVs, they are directed to lysosomal degradation or exosomal release [25]. Technically, the exosomal abscission process is summarized in three-step tethering, docking, and fusion [96]. Membrane tethering, the initial interaction between the MVB membrane and plasma membranes, is mediated by the interaction of Rab GTPases on the plasma membrane with tethering proteins on the MVB membrane [96]. Tethering machinery directs the formation of t-SNARE complexes on the plasma membrane, so triggers the assembly t-SNAREs with v-SNAREs on the surface of MVB and at the end leads to the partial formation of SNARE complex at the site of docking [96]. The balance between lysosomal degradation and exosomal release is controlled by different regulators. Based on studies, the level of Tetraspanin-6 (TSPN6) in the cells plays determining role in the fate of specific cargoes to the degradation of secretory pathways to preserve this balance [97]. Also, the role of TSPN6 has been proved in the exosomal release of (amyloid precursor protein) APP into the HEK293 cells [98]. High cellular levels of TSPN6 via inhibition of Syntenin/Syndecan and Syntenin/CD63 complexes formation prevents exosomal release and promotes lysosomal degradation of cargoes. On the other side low levels of TSPN6 direct the secretory pathway. The diverse effects of TSPN6 on the fate of ILVs possibly are depending on cell types and contexts [99]. The additional transfer of ubiquitinated cargo into ILVs via ESCRT-dependent mechanisms guides ILVs to the lysosomal pathway while the participation of Tetraspanin-dependent mechanisms targets ILVs to the secretory pathway. Noteworthy, ISGylation of peptides along with ubiquitin-like modification orients MVBs to the lysosomal pathway [100]. Unlike this pathway, further activation of RAB31 and interaction with Flotillin belonging to lipid raft microdomains, or TBC1D2B-derived activation of RAB31 (an activator of GTPase) directs MVBs to the secretion pathway rather than lysosomal degradation (Fig. 3A–B) [28].

To promote exosomal abscission, the physical contact of Exo and plasma membrane is mandatory. The promotion of certain factors like actin and actin-regulating proteins, SNAREs, tethering factors, Rabs, and other types of RAS is important [14]. In this regard, cytoskeletal proteins such as actin and actin regulatory proteins (non-muscle myosin II/ROCK1/MLC/actin signaling pathway), microtubules as well as motor molecules (dyneins and kinesins) are involved in the secretion of Exo [101]. Of known proteins involved in docking and loading of MVBs into the inner surface of the plasma membrane, Cortactin and collaboration with Rab27A and coronin 1b promote the fusion of MVBs to membranes [102]. One of the important determinants in the fusion of MVBs to the membrane is associated with P4-ATPase ATP9A located at the inner surface of the plasma membrane and the MVB external surface. The lipid flippase activity of P4-ATPase ATP9A can regulate the recycling/secretion of MVBs [103]. Importantly, the activation of the SNARE complex leads to the fusion of MVBs with the target membrane. These proteins are categorized to v-SNAREs (attached to the vesicles) and t-SNAREs (attached to the target membrane). This complex is composed of syntaxin1 and SNAP25 (t-SNAREs), synaptobrevin (v-SNARE), and regulatory protein synaptotagmin. Depending on the type of organism, cell type, and MVB cargo, different proteins participate in the regulation of Exo secretion [8]. On the other side, oligomerization of the Synaptotagmin regulates the assembly of the SNARE complex at the docking site [104]. These features result in fusion of the MVB membrane with the plasma membrane and at the end release Exo [104]. The homologs of Synaptobrevin/Vesicle-associated membrane proteins (VAMP) participate in the Exo secretion process depending on kind of cells. For example, Vamp7 is involved in the exosomal secretion in the K562 cell line from leukemia cells [105] and YKT6 is involved in the exosomal secretion in the HEK293 cell line from the human embryonic kidney and in the A549 cell line of the human lung cancer cells [104].

Due to the complexity of Exo trafficking and the incorporation of numerous effectors from multiple signaling pathways, it is logical to hypothesize that these types of machinery could be used by numerous transduction cascades [18]. Due to similarities between viruses and the Exo biogenesis system, the replication, and intracellular transfer of viruses could be hijacked by viruses in favor of viral replication and propagation to the other cells [106]. In support of this claim, some reports are demonstrating that viruses use Exos or their intracellular machinery system to spread the infection out of the host cells [107].

## Entry pathway of viruses

**Fig. 4:**
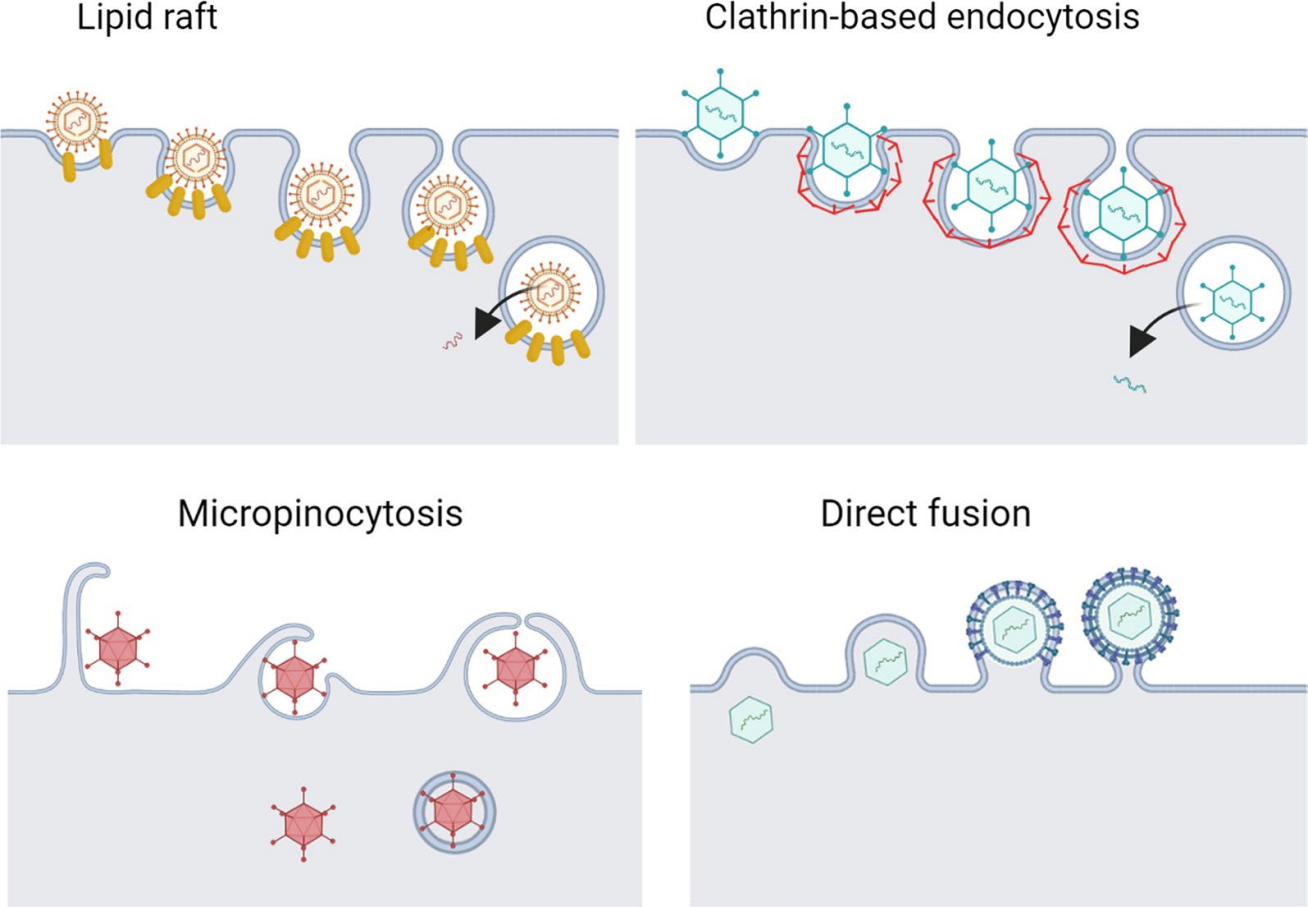
Several mechanisms used by viruses to internalize the host cells

Understanding the mechanisms involved in viral entry seems the first step to answering how the dynamic growth of each virus is associated with the Exo trafficking system [107]. Molecular investigations have revealed that the entry of viral particles into the cells closely depends on overcoming several natural barriers including the plasma membrane, cytoskeletal dam, and intracellular membranes [108]. To circumvent these barriers, viruses commonly exploit direct fusion using endocytic mechanisms [109]. Noteworthy, the internalization strategy of viruses depends on the pH of target cells and several viral properties such as ligands and size [110]. It was suggested that viruses with fusion proteins and certain ligands can enter the host cell using several mechanisms as below (Fig. 4) [111];

### Direct fusion pathway

At physiological pH, some of the enveloped viruses such as human immunodeficiency virus (HIV), and Herpes simplex viruses (HSV) like Epstein Barr Virus (EBV) are transferred into the cytosol using direct fusion [112]. Direct fusion includes three distinct steps as follows; tethering, docking, and further fusion. The latter step can be orchestrated via direct or indirect fusion [113]. Under physiological conditions, viral tethering is initiated by the non-permanent and non-specific attachment of viral ligands to proteoglycans and glycolipids [114]. After that, the docking step is triggered which is followed by the exposure of modules to maintain specific interaction and in the next phase, the fusion of viral particles and cell membrane occurs due to conformational remodeling of ligands and receptors [115]. It has been shown that the involvement of certain signaling pathways can facilitate the procedure of viral entry into the cytosol. For instance, HIV attaches to the cell surface after the interaction of viral fusion glycoprotein namely gp41/120 to the cognate receptor CD4 [116]. In next steps, other receptors like CXCR4 or CCR5 are promoted on the cell surface to perform specific binding and changes in viral glycoprotein for accelerating viral entry [117].

### Endocytic pathways

Endocytic uptake is the collection of biological changes in the host cell membrane which are associated with the formation of curvature endocytic vesicles to carry viral particles into the deeper part of cytosol [118, 119]. It is believed that the integration and involvement of certain signaling cascades are essential to promoting the formation of endocytic vesicles [120]. Of note, lumen acidification in early or late endosomes can speed up virus penetration into the cytosol [121]. The active role of endocytic signaling pathways such as clathrin-dependent endocytosis, micropinocytosis, and lipid raft-dependent mechanisms are integral to the formation of viral endocytic vesicles [122, 123]. Among lipid raft-dependent endocytosis, pathways based on caveolae, flotillin, and GTPase regulators associated with focal adhesion kinase1 (GRAF1) can be exemplified [124].

**Fig. 5:**
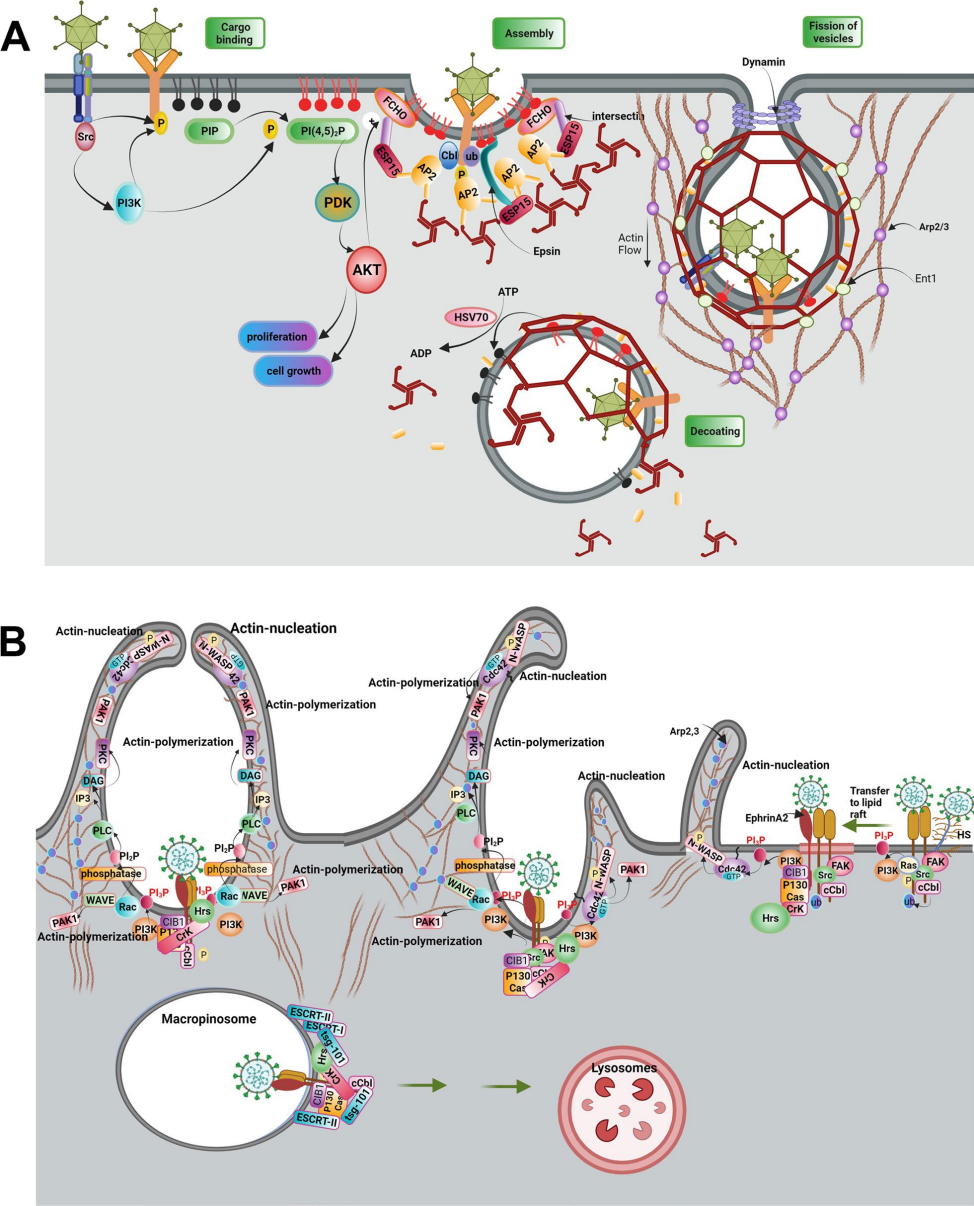
Internalization of the virus using Clathrin-dependent endocytosis (**A**). Virus attachment to a wide variety of cell receptors can induce activation and autophosphorylation of Src. Interaction of Src with cell surface receptors drives phosphorylation of receptors and activation of the PI3K/Akt/mTOR signaling pathway. PI3-K via activation of cCbl (E3ubiquitin ligase) multi-ubiquitylates EGFR, which drives phosphorylation and ubiquitination of receptor, then translocates virus bonded receptors into lipid raft. Ubiquitinated receptor drives phosphorylation and monoubiquitylation of EPS15 and monoubiquitylation of epsin. Epsin is a scaffold protein and binds to PI(4,5)_2_P in lipid rafts, cargo receptors, Esp15, AP2 protein, and clathrin molecules. Furthermore, the interaction of ESP15 with FCHO and intersectin proteins forms a trimeric FCHo/Eps15/ intersectin complex that can interact with AP2. Thus, AP2 is recruited for attachment to the membrane receptors and Clathrin molecules via interaction with FCHo/Eps15/ intersectin complex and Epsin proteins. Nucleation of AP2 can increase the local concentration of PIP2 by incrementing the activity of PIP kinase. Membrane fission of clathrin-coated vesicles is followed by the formation of dynamin helical oligomers at the neck of vesicles. After scission, vesicles are uncoated by the activity of the HSC70 chaperone, which dephosphorylates PI(4,5)P2 to PI4P, at the results clathrin coat is disassembled. Internalization mechanism of the virus by micropinocytosis (**B**). entry of the virus into cells is mediated by the initial attachment of the virus to the heparan sulfate and subsequently, integrins molecules, which is followed by induction of FAK, Src, Ras, and PI3K, signaling molecules. Activation of PI3-K recruits Ub-ligase cCbl which, both monoubiquitinates integrins molecules and leads them into the lipid raft on PM. In lipid rafts virus attached to the ubiquitinated integrins interacts with Ephrin A2 receptor; which results in the recruitment of CIB1 (adaptor protein)-p130Cas (scaffold protein)-Crk (effector protein) molecules to Ephrin A2-integrin -virus complex. CIB1-p130Cas-Crk complex recruits Hrs of ESCRT-0 to the site of macropinocytosis on the PM. Besides activated signaling of Ras and PI3-K induce actin nucleation and polymerization via activation of Cdc42-WASP, Rac1-WAVE complexes, and PAK1. Besides, PIP3 on the PM recruits phospholipase C (PLC), which leads producing of IP3 and diacylglycerol (DAG). DAG, in turn, recruits protein kinase C (PKC) to further promote actin polymerization. After the scission of macropinosomes by CtBP1and/or dynamin (is not shown here) recruitment of Tsg101 by Ephrin A2, c-Cbl, and associated signal molecules, and sequential recruitment of ESCRT I-III complex proteins on the endosome directs macropinosomes to the lysosomal degradation

For the entry of viruses via clathrin-based endocytosis, certain viruses such as Hepatitis B Virus (HBV), SARS-CoV-2, and type 1 HSV can attach to growth factor receptors such as epithelial growth factor receptor (EGFR) [125–127]. Upon viral and receptor interaction, tyrosine residues are phosphorylated in participant receptors followed by ubiquitination via the activity of ubiquitin ligase (Fig. 5A). Ubiquitylated receptors provoke other membrane-associated components such as FCH domain only 1/2 (FCHO1/2); epidermal growth factor receptor substrate 15 (EPS15); and intersectin [128]. The direct interaction of FCHO1/2 and intersection to EGFR recruits adaptor protein-2 (AP-2). In the next step, the activation of phosphatidylinositol phosphate kinase increases local levels of PIP2, leading to the assembly of membrane clathrin [128]. Of note, the polymerization of actin fibers accelerates membrane invagination and localization of clathrin molecules on the surface of vesicles [129]. The procedure of vesicle fission is done by involving dynamin and BAR domain proteins like endophilins and amphiphysins [130]. From the structural view of point, dynamin has GTPase activity that can support dynamin polymerized into the helical structure at the neck of endocytic vesicles, resulting in the disassociation of vesicles from the plasma membrane. Owing to the ATPase activity of head shock cognate protein 70 (HSC70) and hydrolysis phosphate bands in phosphatidylinositol 4, 5-biphosphate [PI (4, 5) P2] by the activity of phospholipase C, clathrin coat is removed from the surface of vesicles [131].

### Micropinocytosis

Micropinocytosis is involved in the entry of certain viruses from different families such as Cytomegaloviridae, Adenoviridae (adenoviruses type 3 and 35), Paramyxoviridae (Nipah virus), Orthomyxoviridae (influenza virus type A), Poxviridae (Vaccinia virus), Togaviridae (Chikungunya virus), and Reoviridae [132, 133]. This process is stimulated following the attachment of the virus to the EGFR co-receptor (Fig. 5B). The activation of tyrosine/serine kinase in receptors provokes and triggers specific downstream effectors such as PI3K, PKC, and Rac1 GTPase. Along with these changes, cytoskeletal remodeling is promoted to the invaginated plasma membrane [132]. Like clathrin-based endocytosis, the GTPase activity of dynamin and kinase activity of CtBP1 help the fission of macropinocytic vesicles [134].

### Lipid raft-dependent endocytosis

#### Caveolae-mediated endocytosis

**Fig. 6:**
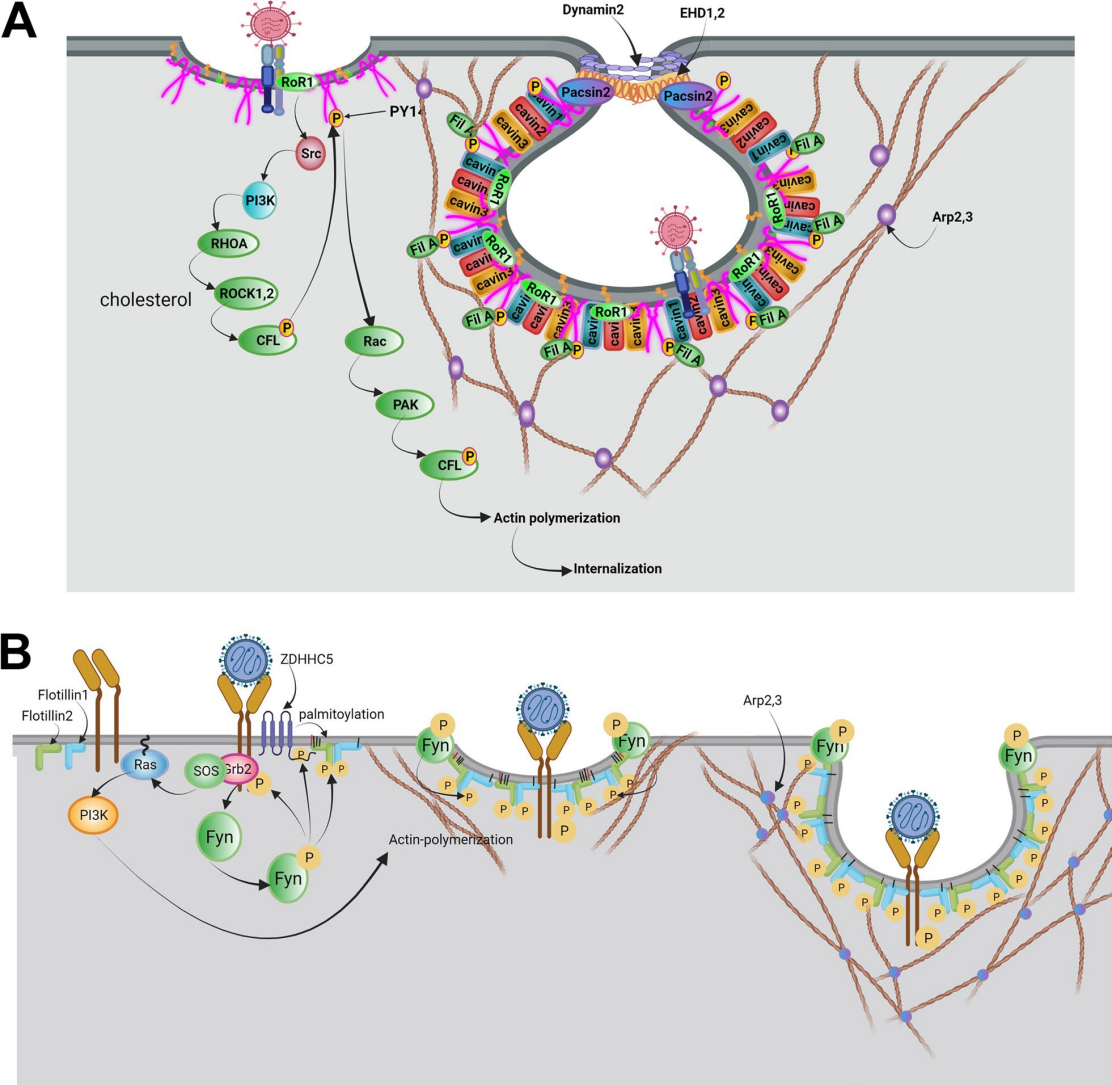
Internalization of the virus using Caveolae-dependent endocytosis (**A**). Attachment of virions to the membrane receptors in membrane microdomains enriched in cholesterol and PtdSer activates receptor tyrosine kinase-like orphan receptor 1 (ROR1) and subsequently directs activation of Src, PI3K, RhoA, ROCK, CFL1 signaling pathway, at the end leads phosphorylation of Caveolin1. ROR1, as a scaffold protein, facilitates the interaction of cavin-1 and phosphorylated Caveolin1 at the PM. Also, Caveolin1 recruits EHD1,2 and Dynamin2 via binding to the Pacsin2, thus leading to the formation of the caveolae neck and session of caveolae vesicles. On the other hand, phosphorylated Caveolin1 leads to actin polymerization via the activation of Rac1, PAK1, and CFL1 signaling pathways. Caveolin1 through binding to Filamin A is associated with actin proteins and regulates caveolae vesicle trafficking. Possible mechanism of virus entry mediated by flotillin-dependent endocytosis (**B**). Initial attachment of the virus to the cell receptor is followed by activation of PI3K, which phosphorylates Fyn kinase, activated Fyn kinase regulates both activation of zDHHC5 and phosphorylation of Flotillins. zDHHC5 via palmitoylation of flotillin1, 2 facilitates their binding to PM. Also, phosphorylated Flotillins induce homo or heterodimerization of flotillins and the formation of invagination vesicles. Besides, activated PI3K leads to actin polymerization and changes the dynamic of the actin cytoskeleton, thus the formation of endocytic vesicles

Some viruses like Simian virus 40 (SV40) exploit caveolae vesicles for cellular entry [135]. From an ultrastructural viewpoint, caveolae are multiple membrane pits that contain high contents of cholesterol and phosphatidylserine [136]. Several factors such as Caveolin1 to 3, Cavin1 to 4, and EHD2, Pacsin2, and receptor tyrosine kinase-like orphan receptor 1 (Rob1) mediate the formation of caveolae (Fig. 6A) [137]. Tyrosine residue 14 of Caveolin-1 is phosphorylated by Src kinase immediately after the attachment of the virus to the plasma membrane [138]. To be specific, Rob1 acts as a docking protein and provides a platform to make complexes by the collaboration of phosphorylated caveolin-1 with cavin [139]. These features in turn lead to the generation of caveolae-coated vesicles. Also, phosphorylated caveolin-1 can interact with cell cytoskeleton via engaging filamin A [140]. By the progression of the signaling pathway, type 2 dynamin accelerates the fission of caveolae-coated vesicles via GTPase activity in an actin-dependent manner [141].

#### Flotillin-dependent endocytosis

Flotillin-dependent endocytosis is involved in the internalization of some viruses such as SARS-COV-2 [142, 143]. The phosphorylation of flotillin by tyrosine kinase activity (Fyn kinase) is touted to be the most important mechanism in flotillin-dependent endocytosis (Fig. 6B). In contrast to different endocytic pathways, the fission and detachment of vesicles from an inner surface of the plasma membrane is done in the absence of dynamin [142, 143].

#### GRAF1-dependent endocytosis

The last mechanism that participates in the viral entry is GRAF1-dependent endocytosis which is exploited by some viral particles such as SARS-COV2, Adeno-associated virus-2 (AAV2), and SV40 [144]. The GTPase activity of GRAF1 and Cdc42 and the presence of GTP binding proteins such as ADP-ribosylation factor 1 (Arf1) help to the formation of tubular invaginations after the stimulation of actin-related proteins-2/3 (ARP2/3) in the absence of dynamin [145, 146].

### Shared types of machinery between Exo biogenesis and viral replication

**Fig. 7:**
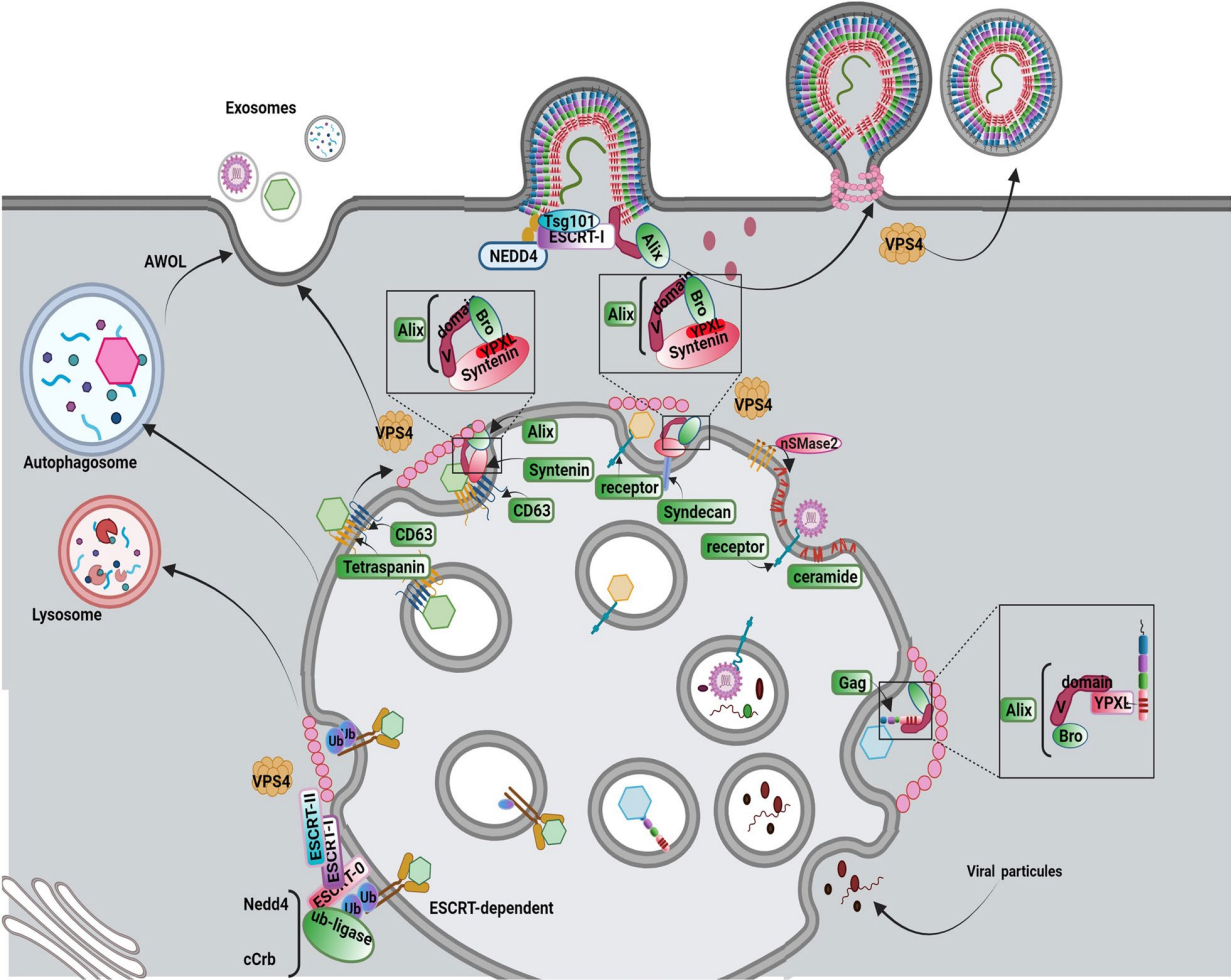
Hijacking mechanisms Cellular adaptor proteins and complexes for promoting viral infection. There are several budding systems for the release of virions. Different enveloped viruses are driven toward PM and hijack the ESCRT pathway via mimic of interactions between cellular adaptors and ESCRT factors. Furthermore, virions invaginate into the MVB and are led to lysosomal degradation or hijack exosomal and autophagosomal pathways. Budding into the MVB is mediated by the hijacking of the ESCRT pathway via attachment to the ubiquitinated viral receptors and finally lysosomal degradation. Also, the entry of virions into the secretion pathway is mediated by the attachment of virions to CD63 and recruitment of the CD63-Syntenin-Alix pathway. In addition to the binding of the virus to the host endosomal receptors and lead formation of ILV by Syndecan-Syntenin-Alix, and formation of ceramide by nSMnase induce. Besides, capsid proteins of virion recruit Alix to the PM and lead assembly of ESCRT-III and formation of virion vesicles. Hijacking Alix protein can promote viral ILV budding and exosomal pathway. ILVs containing LC3 form autophagosome-like vesicles which mediate the exit of the virus through autophagosomal vesicles or AWOL

Exo can reflect instant intracellular changes and pathological conditions like viral infections and anaplastic modification after release into the ECM [11]. Whether the virions have been educated to intentionally exploit Exo trafficking machinery for transmission or the existence of different shared biogenesis systems between Exo machinery and viral replication pathway leads to accidental Exo-based delivery of viruses needs further explanation. To date, the possible positive and negative effects of viral entry on Exo biogenesis and abscission rate have not been completely addressed yet. Based on some evidence, viruses have the potential to incorporate several effectors within Exo biogenesis [147]. It is thought that common pathway between virus replication with Exo biogenesis can accelerate viral replication and abscission [147, 148]. Additionally, the decoration of viruses with host cell lipid layer helps them to enter the other cells using Exo ligands and escape from the direct and indirect activity of immune cells. On the other hand, the uptake of Exo containing viral particles by antigen presenting cells can facilitate antigen-presenting procedures [147, 148]. Therefore, the exosomal delivery of certain viruses by Exo can act as a two-edged sword in the dynamic activity of viral infections (Fig. 7).

Contrary to the release of lytic viruses (non-permissive viruses), the exploitation of Exo biogenesis machinery by non-lytic viruses is mighty in the assembly and release steps using two distinct approaches. As above-mentioned, certain viruses mainly enveloped like enveloped virions can hijack numerous effectors related to plasma membrane fission for internalization and packaging. Regarding the existence of reverse-topology plasma membrane fission and the inevitable role of ESCRT machinery in this procedure, it is thought that ESCRT machinery can mediate, in part but not completely, the release of enveloped viruses from host cells. From a molecular structure view of point, the outward fission of the plasma membrane is done by the activity of ESCRT machinery subsets such as TSG101 of ESCRT-I, ALIX, and NEDD4L [149]. Of course, this mechanism is not the only pathway for the exosomal efflux of viruses. It is suggested that some viruses can enter MVBs in the stage of ILVs formation. This strategy can lead to two separate destinies. On one hand, some fraction of MVBs and late endosomes are directed toward enzymatic digestion by lysosomes which can confine further propagation of viruses. On the other hand, the fusion of MVBs and late endosomes to the plasma membrane can facilitate viral efflux and reach remote sites [147]. It is possible that specific components of viruses such as proteins, and nucleic acids are sorted into Exo lumen or entire viral particles (such as virions) can be transmitted to the other cells. Therefore, it is noteworthy to mention that Exo signaling pathway provides several machinery systems which are critical for viral infection and propagation. In support of this notion, the existence of some viruses such as HIV, HSV-1, rotaviruses, Langat virus, and noroviruses have been identified inside the lumen of released Exo [150]. Along with this statement, viral components of other virus types such as HIV, EBV, CMV, HSV-1, Kaposi's sarcoma-associated herpes virus (KSHV), and coronaviruses can be detected inside the Exo particles.

#### Invagination of viral particles using ESCRT complex

Viruses can hijack factors related to the ESCRT pathway by providing viral structural proteins that mimic natural ligand activity. To this end, virus-associated structural proteins can recruit the ESCRT complex components using certain motifs that can interact with cell surface receptors. P (T/S) AP, YPXL, and PPXY are such motifs within the viral structure that bind with surface receptors [151]. It has been indicated that the activation of P (T/S)AP motifs in the HRS subunit of ESCRT-0 can recruit ESCRT-1 TSG101 via binding of the P(T/S) AP motif with the UEV domain of TSG101 [152]. The similar structure of the P (T/S) AP motif has been indicated within viral Gag protein from Retroviridiae (such as HIV and murine leukemia virus), Z proteins of Arenaviridae (Lassa virus and lymphocytic choriomeningitis virus), VP40 proteins of Filoviridae (Ebola virus and Marburg virus), and M (Matrix) proteins of Rhabdoviridae (vesicular stomatitis virus and rabies virus) [153–157]. These factors can activate TGG101, leading to the participation of the ESCRT complex at the site of virus entry [149]. Within the cells, the YPX_n_L motif of syntenin can interact with the V-domain of Alix. It is believed that Alix actively is involved in the MVB pathway [158]. Many viruses belong to different families like Retroviridiae, Arenaviridae, Hepadnavirideae, Herpesviridae, Flaviviridae, Filoviridae, Paramyxoviridae, and Tombusviridae are potent to activate such mechanisms for viral particle entry [156, 159, 160]. The interaction of the viral YP_n_XL, motif with the V-domain of Alix can be exploited in favor of viral entry [161]. PPXY is another motif with the ability to interact with WW and HECT domains of the NEDD4 family and E3 ubiquitin ligases. The interaction promotes the ubiquitylation using NEDD4, as an ESCRT auxiliary protein, leading to the sorting of proteins into the MVBs [162]. Of note, a PPXY-like motif exists in several viruses from different families such as Retroviridiae, Rhabdoviridae [157], Arenaviridae [153], Filoviridae [156], Reoviridae [149], Hepadnavirideae [149], and Paramyxoviridae [159]. Similarly, the interaction of PPXY-NEDD4 increases the budding of the virus into the MVBs. Notably, in specific virus types such as HIV, the existence of several motifs in Gag can recall different adaptor proteins of the ESCRT system for the promotion of the budding procedure [163].

#### Viral efflux via ESCRT-dependent exosomal system

It is thought that both enveloped and non-enveloped viruses can be internalized into the releasing Exo via the recruitment of ESRT components or associated proteins [164, 165]. Experiments have revealed that hepatitis A virus (HAV) can be detected in the circulation in two distinct forms; naked non-enveloped capsid and quasi–enveloped virion (eHAV) [166]. The second form is resistant to host immune system activity. The release of eHAV can be done via the ESCRT subsets such as HRS and TSG101 and ESCRT-associated proteins such as Alix and VPS4. In support of this declaration, the suppression of HRS and TSG101 did not completely affect eHAV efflux while the inhibition of VPS4B and Alix led to the accumulation of eHAV inside the host cells [166]. In contrast to the eHAV, suppression of HRS significantly diminishes the exit of hepatitis C virus (HCV) release and the number of viral particles increased inside the cytosol [167]. Molecular analyses have revealed that ubiquitination of HCV core protein by E6AP (E3 ubiquitin ligase) leads to the interaction of the virus with HRS and subsequent cloaking after entrance into the endosomal membranes. According to the data, it is suggested that HCV virions can be sorted into the releasing MVBs [168]. The inhibition of MVB formation using certain chemicals such as U18666A aborts HCV release without any modification in the intracellular assembly of viral particles [167].

#### Viruses efflux via ESCRT-independent exosomal system

Viruses can also exploit ESCRT-independent pathways to assemble and enter into the MVBs [169]. This approach is not affected after the suppression of ATPase activity in Vps4. Whether and how different viruses can use different exosomal pathways for releasing efflux has not been fully addressed. It has been shown that several viruses belonging to the Picornaviridae family such as enteroviruses can use plasma membrane-derived and endosomal vesicles for assembly and propagation (Table 1) [169]. Among different types of enteroviruses, poliovirus can spread using autophagosome-like vesicles. This phenomenon is also known as autophagosome-mediated exit without lysis (AWOL). Other enteroviruses such as Coxsackievirus B3 (CVB3) use an AWOL-like mechanism for virion release. Besides, the efflux of CVB3 particles via microvesicles has also been proved using fluorescent timers [170]. Images provided by transmission electron microscopy have indicated the release of CVB3 virions from the host cells via engaging microvesicles. Enterovirus 71 (EV71) is another enterovirus disseminated using EVs [171]. Molecular investigations have supported the sorting of VP1 protein and RNA of EV71 virion into the EVs. Upon the completion of the efflux procedure using EVs, viruses can infect the acceptor cells. For instance, it has been shown that HPV-containing Exo can affect human neuroblastoma cell lines [171]. Data suggest the participation of different exosomal pathways for the assembly and propagation of certain virus types [172]. For example, the dissemination of the human herpes virus (HHV-6) is done by Exo. During the maturation and assembly of the HHV-6, both, Trans-Golgi network vesicular and tubular structures can be discriminated. Proteomic investigations have shown that these particles express both tetraspanins such as CD63 and HHV-6 virion proteins. Interestingly, co-localization of HHV-6 envelope protein (gB) and CD63 has been indicated in late endosomes juxtaposed to the nucleus [172]. As above-mentioned, the decoration of viral particles such as rotavirus and norovirus inside secretory vesicles can increase the possibility of infection rate [173]. Like different viruses, entrap of norovirus inside CD9^+^, CD81^+^, and CD63^+^ Exo has been indicated [174]. The suppression of ceramide synthesis after inhibition of sphingomyelinases via GW4869 reduces the exosomal efflux of noroviruses [169]. Based on microfluidic tracking analysis, it has been shown that the amount of rotavirus-containing vesicles and the number of vesicle proteins increased after rotavirus infection. Both microvesicles and Exo are involved in the transmission of rotavirus particles [150]. A load of viral particles can be done in terms of Exo surface or lumen. Of note, the attachment virion to the surface of EVs is not ordinary and was seen in some types of viruses like rotaviruses, polyomaviruses, HSV-1, and adenoviruses. The existence of VP4 and VP8 is suitable evidence for exosomal delivery of rotavirus [150]. Within the cells, the physical interaction of viral factor NSP4 with endoplasmic reticulum DLP provides a platform to increase the interaction of viral VP4 with lipid raft domains, resulting in the penetration of viral particles into the Exo and microvesicles [175]. In some circumstances, the completion of viral assembly is associated with the activity of Exo. For example, the transmission of the Ebola virus (EBOV) glycoprotein (GP), VP40, and nucleoprotein (NP) depend on Exo and microvesicle activity [176]. It is thought that three mechanisms exist inside Exo for the internalization of EBOV proteins [177]. First, EBO virion can enter the cell after the formation of early endosomes. Upon the release of viral genetic elements from endosomes, proteins such as VP40 and GP do not fully exist and are stable during Exo biogenesis [177]. In the second mechanism, EBOV proteins are transferred into the ILV lumen inside the MVBs. The ubiquitination of VP40 promotes its entry into the Exo via the collaboration of the ESCRT complex. Along with these activities, GP can be guided into Trans-Golgi vesicles and viral RNA (NP) is directed into Exo via unknown mechanisms. In the latter mechanism, viral particles can be released into the ECM after outward budding from plasma membrane. Noteworthy, viral protein VP40 is attached to the vesicle surface while both NP and RNA are located at inner surface of vesicle membrane [177].

**Table 1:** Mechanisms used by several virus types to release from the host cells

Type of virus	Family	Intracellular transfer	Hijacking mechanism	Releasing mechanisms	Reference
*Enveloped DNA viruses*					
Epstein-Barr Virus	Herpesviridae	ESCRT-dependent and ESCRT-independent pathways	TSG101, CD63, Syntenin‑1, Alix, and lipid rafts	Late endosomes	[195, 196, 238]
Kaposi's sarcoma-associated herpesvirus	Herpesviridae	ESCRT-dependent and ESCRT-independent pathways	Viral LMP1	Late endosomes	[182, 239]
Human Herpes virus-6	Herpesviridae	ESCRT-independent pathway	CD63 and viral gB	Late endosomes	[172]
Human Cytomegalovirus	Herpesviridae	ESCRT-independent, Vamp3-dependent pathways	VAMP3, viral pUL71 and UL99	Late endosomes	[197, 240]
Herpes simplex virus 1	Herpesviridae	ESCRT-dependent pathway	ESCRT-III	Microvesicular budding, Autophagosome-mediated exit without lysis, Late endosomes	[241, 242]
*Non-enveloped DNA viruses*					
Adenoviruses 2 and 5	Adenoviridae	ESCRT-independent pathway	Exosomal CD46, Lamp2c	Late endosomes, Microvesicular budding	[181, 243, 244]
Hepatitis B virus	Hepadnavirideae	ESCRT-dependent pathway	α-taxilin, viral LHBs, TSG101, 2-adaptin, Nedd4	Late endosomes, Microvesicular budding	[245]
*Enveloped RNA viruses*					
Japanese encephalitis virus	Flaviviridae	ESCRT-dependent pathway	viral NS3, TSG101	Late endosomes	[246, 247]
Yellow fever virus	Flaviviridae	ESCRT-dependent pathway	Viral NS3 protein, Alix	Late endosomes	[248, 249]
Dengue virus	Flaviviridae	ESCRT-dependent pathway	Viral NS3, Alix, viral protein E, Tsp29Fb	Late endosomes	[186, 187, 249, 250]
West Nile virus	Flaviviridae	ESCRT-dependent pathway	Viral NS3, Alix and ESCRT	Late endosomes	[249]
Zika virus	Flaviviridae	ESCRT-dependent pathway	Viral NS3 protein interacts with Alix	Late endosomes	[249, 251]
Tick-borne encephalitis	Flaviviridae	ESCRT-dependent pathway	ND	Late endosomes	[249]
Hepatitis C Virus	Flaviviridae	ESCRT-dependent pathway	Viral NS2 and NS5A proteins, Ago2, HSP90, and CD81	Late endosomes	[252–254]
Human immunodeficiency virus-1	Retroviridiae	ESCRT-dependent pathway	Viral Nef, Alix, viral Gag, and Syntenin	Late endosomes	[161, 191, 255, 256]
Equine Infectious Anemia Virus	Retroviridiae	ESCRT-dependent pathway	Viral Gag, Alix	Late endosomes TGN is the assembly site	[257]
Feline immunodeficiency virus	Retroviridiae	ESCRT-dependent pathway	Viral Gag, and Alix	Microvesicular budding	[258, 259]
Human immunodeficiency virus-2	Retroviridiae	ESCRT-dependent pathway	Viral Nef, Alix and viral Gag, and TSG-101	Late endosomes, Microvesicular budding	[260, 261]
Mouse Mammary Tumor Virus	Retroviridiae	ESCRT-dependent pathway	Viral Gag and NEDD4	ND	[262]
Murine Leukemia Virus	Retroviridiae	ESCRT-dependent pathway	Viral Gag and WWP1	Late endosomes, Microvesicular budding	[260, 263, 264]
Porcine endogenous retrovirus	Retroviridiae	ESCRT-dependent pathway	Viral Gag, and WWP2	Late endosomes	[265]
Prototypic foamy viruses	Retroviridiae	ESCRT-dependent pathway	Viral Gag, TSG-101, and PPPI	Late endosomes, Microvesicular budding, trans-Golgi network	[266, 267]
Mason–Pfizer monkey virus	Retroviridiae	ESCRT-dependent pathway	Viral Gag, TSG-101 and NEDD4	Microvesicular budding	[268, 269]
Human T cell leukemia virus type 1	Retroviridiae	ESCRT-dependent pathway	Viral Gag, TSG-101 and NEDD4	Late endosomes	[260, 270]
Human endogenous retrovirus-K	Retroviridiae	ESCRT-dependent pathway	Viral Gag, TSG-101	Late endosomes	[271, 272]
Rous sarcoma virus	Retroviridiae	ESCRT-dependent pathway	Gag, NEDD4 and Alix	Late endosomes	[273, 274]
Respiratory syncytial virus	Pneumoviridae	ESCRT-independent pathway	ND	Late endosomes	[46]
Human Pegivirus, GB virus C	Flaviviridae	ESCRT-dependent pathway	hnRNPA2B1 protein	Late endosomes	[275, 276]
Ebola virus	Flaviviridae	ESCRT-dependent pathway	Viral VP40, TSG-101, NEDD4, and VP24	Late endosomes, Microvesicular budding	[277, 278]
Marburg virus	Flaviviridae	ESCRT-dependent pathway	TSG-101, Viral VP40 and NEDD4	Microvesicular budding	[279–281]
Lassa fever virus	Arenaviridae	ESCRT-dependent pathway	Z protein and TSG-101	Microvesicular budding	[282, 283]
Rabies Virus	Rhabdoviridae	ESCRT-dependent pathway	Viral M protein, ESCRTI, Alix, ESCRT III and VPS4	Microvesicular budding	[284, 285]
Vesicular stomatitis virus	Rhabdoviridae	ESCRT-dependent pathway	Viral M protein, TSG-101, and NEDD4	Microvesicular budding	[286]
Parainfluenza virus 5	Paramyxoviridae	ESCRT-dependent pathway	Viral M protein, and VPS4 ATPase	Microvesicular budding	[287]
Parainfluenza virus 3	Paramyxoviridae	ESCRT-dependent pathway	Viral M protein	Microvesicular budding	[288]
Newcastle disease	Paramyxoviridae	ESCRT-dependent pathway	Viral M protein and VPS4 ATPase	Microvesicular budding	[289]
Mumps virus	Paramyxoviridae	ESCRT-dependent pathway	Viral M protein and VPS4 ATPase	Microvesicular budding	[290]
Hepatitis E virus	Hepeviridae	ESCRT-dependent pathway	Viral pORF3, and TSG-101	Late endosomes	[291, 292]
*Non-enveloped RNA viruses*					
Hepatitis A virus	Picornaviridae	ESCRT-dependent pathway	Viral VP2, Alix and VPS4B	Late endosomes	[166, 293]
Coxsackievirus B	Picornaviridae	ESCRT-dependent pathway	LC3	Microvesicular budding, Autophagosome-mediated exit without lysis and	[294]
Poliovirus	Picornaviridae	ESCRT-dependent and independent pathways	Alix	Microvesicular budding, Autophagosome-mediated exit without lysis and	[165]
Enterovirus 71	Picornaviridae	ESCRT-dependent pathway	ND	Late endosomes	[295–297]
Reovirus	Reoviridae	ESCRT-dependent pathway	Rab5, Viral capsid protein P2	Late endosomes, modified lysosomal organelle act as transporter virions to the plasma membrane	[184, 298]
Rotavirus	Reoviridae	Probably ESCRT-dependent pathway	Probably VP4 interacts with lipid Rafts	Microvesicular budding	[150]
Norovirus	Caliciviridae	Probably ESCRT-dependent pathway	Viral VP1, Phosphatidylserine lipid	Late endosomes	[174]

It should not be forgotten that the Trojan activity of each virus can be used for several therapeutic purposes [178]. For instance, oncolytic adenovirus-loaded EVs can be used for successful transfection and genetic manipulation of cancer cells [179]. Further propagation and viral spread are done by different proposed mechanisms. Viral bodies can be externalized from host cells after the formation of membrane-associated microvesicles. The attachment of viral components inside the lumen and surface of MVBs has touted another underlying mechanism for the efflux of adenoviral infection out of the host cells [180]. Finally, the invagination of ILVs inside the MVB lumen can also help viral propagation. As described in Exo signaling pathway, some of MVBs may be directed toward the lysosomal degradation procedure [181]. Similarly, the transfer of KSHV genetic materials from cell to cell has been documented using Exo [182]. Even though, the transfer of Exo-containing KSHV RNA can dictate specific metabolic conditions in recipient cells. For example, upon the entry of KSHV-loaded Exo, the respiration capacity of mitochondria is diminished coinciding with the induction hypoxic condition. One reason would be that KSHV-infected cells produce Exo which contains a large amount of lactate dehydrogenase, resulting in the shift of oxidative phosphorylation toward glycolysis [182]. Because of the diversity in viral activity and the existence of different pathogenic molecules in the structure of viruses, it is logical to hypothesize that viruses can, in part, exploit different parts of the Exo signaling pathway. Previous studies confirmed the critical role of Exo in the dissemination of non-lytic human reoviruses from host cells to neighbor cells. This virus is transferred as virion bodies by complete incorporation into an exosomal system [183]. Considering the phylogenetic association of the Exo-related pathway, the mechanism of viral hijack has some similarities between the species. Reoviruses can maintain specific interaction between the nucleocapsid domain and intracellular Rab5, participating in virus packaging via releasing Exo [184]. Concerning transport activity, reoviruses can also bind to specific motifs RGD and KGE, and NPXY of λ2 nucleocapsid to the carboxylic domain of β1 integrin and enter the endosomes [183, 185]. In another study, it was shown that the entry and release of Dengue virus (DENV), belonging to Flaviviridae and transmitted by mosquitoes, can be done by Exo. Analyses have confirmed the entry of DENV into the Exo can increase exosomal diameter compared to intact Exo. Interestingly, these Exo can fit complete and fragmented viral components [186]. It is believed that human CD63 ortholog namely Tsp29Fb, a member of the tetraspanin complex, is responsible for viral loading into the Exo lumen via direct interaction of viral E protein with Tsp29Fb [187]. Within the pulmonary niche, the influenza virus can be easily transmitted between epithelial cells via Exo. These Exo can harbor viral antigens, proteins, and genetic materials in addition to inflammatory factors [188, 189]. Evidence points to the fact under specific conditions the dynamic viral infection and Exo biogenesis are closely interconnected [190]. For example, the infection of cells with the rabies virus can reduce the transit time of Exo inside the cytosol, leading to an accelerated Exo release. The inhibition of Exo secretory mechanism using certain chemical s and GW4869 and si-Rab27a leads to apparent reduction of Exo biogenesis and elimination of viral RNA [190].

HIV is another virus that uses several dissemination mechanisms. This virus provides motifs Gag protein which is actively involved in the formation of budding from the plasma membrane. Besides, the invagination of early endosomes and formation of ILVs are integral to the transport of HIV Nef protein and vmiRTAR, and finally, the release of viral Tat protein via budding and direct diffusion can help viral particle dissemination [191]. Using an Exo-based delivery system, HIV can be resistant to the host immune system for a long time. The replication of HIV has not been shown. Interestingly, HIV-loaded Exo have significant fusion capacity due to the fusogenic activity of the Tat factor. It seems that the internalization of HIV-loaded Exo can be promoted via ESCRT-dependent and associated proteins. In Nef containing Exo, ALIX is provoked to internalize the Exo cargo. It has been shown that Nef can regulate the sorting of RNAs into the Exo lumen. For example, the activity of RNA-TAR (miRTAR) is regulated by Nef, leading to rapid viral propagation inside the host cells [192, 193]. Epstein–Barr virus (EBV) belongs to the oncovirus with tumorigenic activity [194]. Notably, the existence of specific microenvironment conditions such as hypoxia and low-pH rates increase Exo and propagation of EBV [195]. The procedure of EBV-encoded RNA (EBER) and LMP-1 loading into the Exo lumen is associated with ESCRT complex activity. Other associated factors such as HRS, TSG101, CD63, Syntenin-1, and Alix facilitate sorting into Exo [196]. To be specific, the direct interaction of LMP-1 with CD63 and/or physical attachment of LMP-1 to lipid rafts promotes cargo sorting into Exo and spread viral particles in immune cells [196]. Proteomic analysis of Exo released from cells infected with HCMV showed the common membrane origin between the virions and Exo. Data indicated that viruses use Exo ESCRT machinery for assembly and transmission with the collaboration of VMP3 [197].

Angiotensin-converting enzyme 2 (ACE2) is a membrane-bound receptor that can attach to spike ligands belonging to the Coronaviridae family. It is thought that the participation of CD9, a tetraspanin component, can facilitate the entry of certain coronaviruses into the cells. CD9 and dipeptidyl peptidase 4 (DPP4) in collaboration with transmembrane protease serine subtype 2 (TMPRSS2) act as linkers for the physical attachment of spike proteins to the host cell membrane, leading to the fusion of the virus with the host cells [198]. Regarding the critical role of CD9 in the sorting into Exo, the existence of CD9 in released Exo is a common feature of virus-infected cells which use the Exo signaling pathway [88]. As mentioned before, rotaviruses can exploit the Trans-Golgi apparatus for propagation and dissemination. On this basis, the close interaction of GBF1 with NSP4 induces trimerization of VP7, and the association of assembly protein VP7 and spike protein VP4 [175]. In the next steps, VP4 is localized to lipid raft microdomains Trans-Golgi apparatus, leading to rotavirus capsid assembly. Simultaneously, the formation of NSP4/VP7/VP4 in the lipid raft microdomains or ERGIC compartments is initiated [199]. It seems that ERGIC35 can promote the entry of VP4 (spike protein) and VP7 (assembly protein) into the vesicles [200]. The critical role of lipid rafts domains with high levels of cholesterol and sphingolipids is integral to cargo sorting [201, 202].

It is believed that Tetraspanin microdomains of CD9, CD63, CD81, and/or CD82 can provide large fusion areas or budding platforms [203]. Previously, the accumulation of components of the ESCRT complex for the efflux of viral particles has been indicated at the site of budding after infection with HIV and HAV [204–206]. In line with this claim, the application of anti-Tetraspanin antibodies can significantly alter viral internalization or fusion [207]. As such, these antibodies are potent enough to inhibit the coronavirus and low-pathogen influenza virus infection [208].

Within the host cells, several signaling cascades can be exploited by viruses. For example, viruses like influenza, HSV1, HIV1, etc. can hijack PI3K/Akt axis for effective viral entry [209]. Unlike these viruses, certain viruses infecting epithelial cells such as porcine sapovirus, SARS COV-2, and other viruses use can tight junctions (TJs) and relevant downstream RhoA/ROCK/pMLC signaling pathways to access co-receptors required for viral entry [210–212]. In this line, the interaction of viral particles with cell surface receptors activates Src kinases and induces downstream signaling pathways. It was suggested that Src associates with cell surface receptors directly, G-protein-coupled receptors (GPCRs), and receptors of tyrosine kinases (RTKs), and indirectly, integrin-mediated by focal adhesion kinase (FAK), leading to the phosphorylation of Rho GTPases and PI3K [209, 213]. As a correlate, the inhibition of EGFR and PI3K phosphorylation plus PIP2 can reduce viral uptake [214]. Src can associate lipid rafts and interfere in the biogenesis and secretion of Exos. This activity is done by the phosphorylation of the cytosolic domain of syndecan and syntenin tyrosine 46 via Src [215]. Besides, the close interaction of Src with Alix induces ILV formation [216].

## Challenges and opportunities associated with exosomal delivery of viruses

**Fig. 8:**
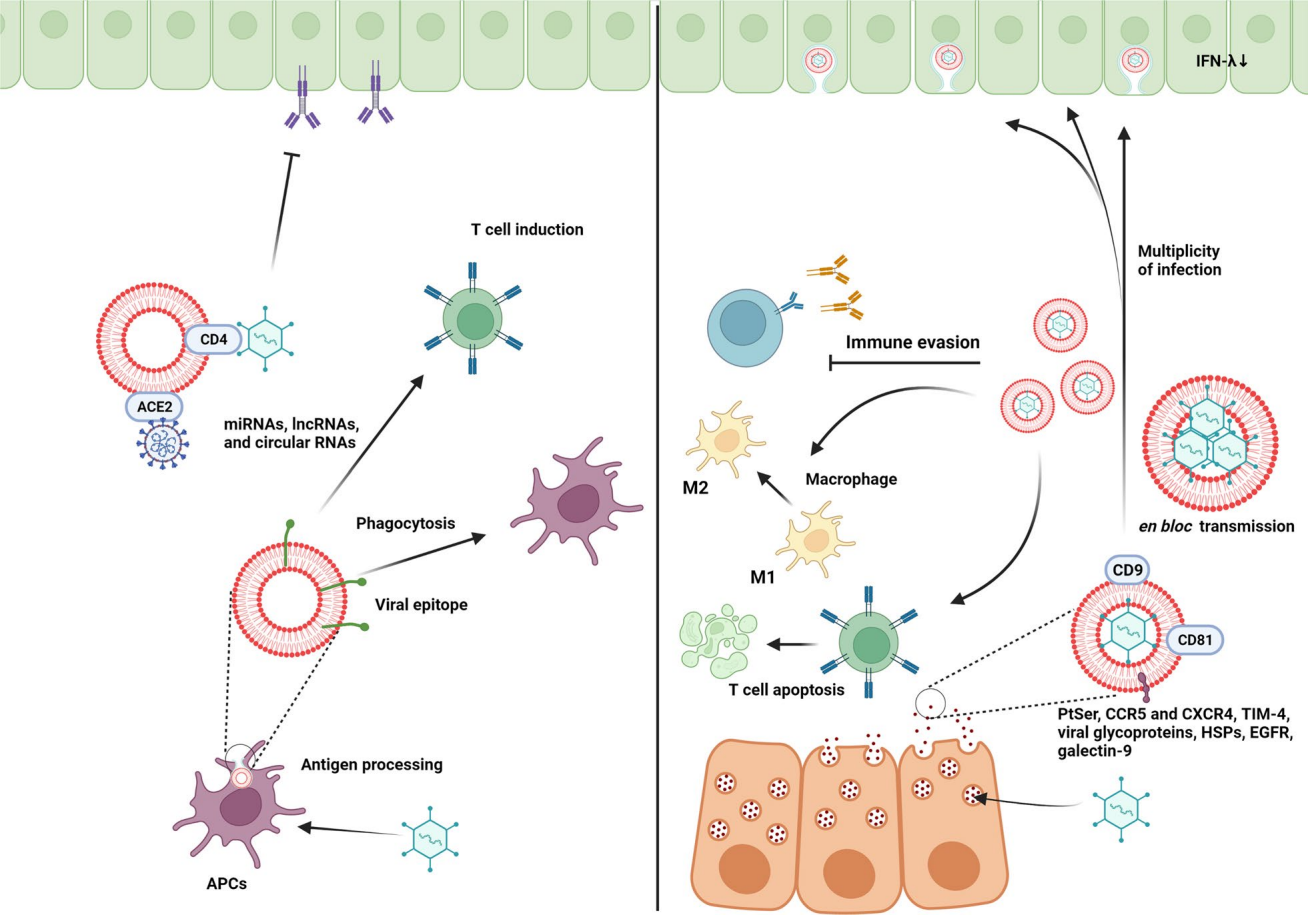
Opposing effects of Exo on viral infectivity and immunity

As previously mentioned, viruses can replicate following the entry into the host cells, leading to cytopathic effects and tissue damage. Within the body, the activation of the host immune system control virus replication and transmission of infectious particles at early steps [217]. According to some data, specific types of viruses can hijack multiple secretion pathways of the host cells such as Exo biogenesis machinery. Thus, one could hypothesize that the formation of an exosomal lipid membrane around viral particles can act as a natural barrier and prevent direct contact of immune cells with viral components [218]. Given the fact that Exo can distribute within the whole body, it is thought that viruses can easily spread to remote sites using Exo as a biological transport system. To be specific, the increase of Exo secretion accelerates the propagation of viral bodies from the secreting cells to acceptor cells (Fig. 8; Table 2) [219]. In support of this notion, it has been shown that EVs can release CCR5 and CXCR4 which act as receptors and co-receptors for the entry of HIV into the target cells. The lack of these factors limits the transmission of this virus into non-talent cells. Like the HIV envelope, the existence of phosphatidylserine receptor TIM-4 on the Exo surface can increase the HIV transfection rate [220]. Logically, the simultaneous transmission of the viral body with signaling molecules increases the possibility of infection in non-talent receptor-negative cells, leading to accidental viral infection via the Exo machinery. In non-envelop viruses such as HAV and hepatitis E virus (HEV), Exo can constitute a pseudo-envelope around these particles and act as a Trojan horse for massive propagation [218]. Therefore, it seems that the close interaction of viral components with an exosomal delivery system can lead to a multiplicity of infections. In an experiment, the production of engineered Exo containing high levels of 29-mer peptide, rabies virus glycoprotein, increased the interaction of these Exo with acetylcholine receptors in neural cells [7]. Interestingly, EVs transmit viruses as single particles or clustered aggregates linked tightly via spatiotemporal coupling such as enteroviruses, leading to *en bloc* transmission of viruses [221]. Ultrastructural imaging and molecular investigations have revealed that Exo are eligible bioshuttles to carry infectious virion-free naked genomes or proteins into the uninfected cells. For example, exosomal transfer of HCV replicon in in vitro conditions has led to cell infection with less efficiency [222]. Despite these possibilities, communications and interaction between the cells via Exo is more complicated rather than a simple transfer procedure.

**Table 2:** Mechanisms related to Exo-based viral immunity and tolerance

Virus type	Immunity mechanisms	Outcome
Various viruses	Exosomal miR-146a↑ and miR-148a↑	Inflammatory response ↑
Rabies virus	Exosomal miR-423-5p↑	IFNβ-related signaling pathway↑, Early-stage viral immunity↑
HCV	Exosomal miR-21↑, miR-29a↑, miR-19a↑, and miR-192	Immune system regulation↑
HBV	Exosomal miR-192↑, miR-21↑, miR-215↑, miR-221↑, and miR-222↑, miR-29a↑, HBx↑, HBV pre-genomic RNA↑	Immune system regulation↑, Il-12↓, Il-6↓, PDL-1↓
EBV	Exosomal miR-21↑, miR-29a↑, miR-BART3↑, miR-BHRF1-1↑, LMP1 ↑	Macrophage IL-12↓, NK cells activity ↓, modulation of the immune response, T cell proliferation↓
Dengue virus-2	Viral NS3 protein↑ and several special miRNAs↑	Early defense program↑
HSV-1	Exosomal miR-H28↑ and miR-H29↑, glycoprotein B↑	Regulation of immune function
Enterovirus 71	Exosomal miR-146a↑	IFN-related pathway in immune cells↓
Ebola virus	Exosomal VP40 NP↑, Glycoprotein↑	Apoptosis of T, B cells, and monocytes↑
Japanese encephalitis virus	Exosomal miR-let-7a/b↑	Apoptotic pathways↑
HIV	Exosomal miR-16↓, miR-125b↓, miR-146a↑, miR-21↑, Nef↑	Immune response↑, pro-inflammatory cytokines↑, activation of monocytes and macrophages↑
Polyomavirus 2	Exosomal jcv-miR-J1-5p↑, jcv-miR-J1-3p↑	large T antigen↓, UL16-binding protein 3↓, NK cell activity↓, viral infection ↑
Rhinovirus	Exosomal miR-155↑, Tenascin-c↑	Pro-inflammatory cytokines↑
Influenza A virus	Exosomal miR-17-5p↑, miR-483-3p, 5p↑, mir-374-5p↑, and miR-446i↑, miR-451a↑, miR-5100↑ and miR-7704↑	miR-17-5p leads to antiviral factor Mx1↓, and viral infection↑ The induction of miR-483-3p, 5p↑, mir-374-5p↑, and miR-446i↑, miR-451a↑, miR-5100↑ and miR-7704↑ can contribute to reduction of viral infection via IFN-β↑, IL-6↑, TNF-α↑, C–C motif chemokine ligand 2 (CCL2)↑, SP100↑

Although explored to a lesser extent, Exo can protect viruses against several immune cell reactions via distinct mechanisms. Membrane-cloaked viruses can efficiently evade neutralizing antibodies [223]. Besides non-lytic spread of viruses from the host cells reduce pro-inflammatory responses and the possibility of necrotic changes and decoration of the Exo surface with certain factors reduces immune cell reactivity [221]. It is thought that the existence of MHC molecules on the exosomal surface diminishes the presentation of viral antigens to the APCs [224]. In addition to the critical role of Exo in hiding viral antigens from the immune system, the existence of distinct biomolecules in the exosomal lumen and membrane can in turn weaken immune cell response. The molecular identity of Exo exhibits certain factors such as HSPs (HSP72) with the ability to activate STAT-3, leading to immunosuppression via reduced M1 to M2 macrophage polarization [225, 226]. Besides, the existence of several growth factors and receptors such as EGFR can lead to the desensitization of macrophages against particles via engaging MEKK2 and IRF3 [227]. Likewise, EBV-infected cells release Exo which are enriched in galectin-9. This factor can promote apoptotic changes in type 1 T helper lymphocytes [228]. Other viral genetic pools transferred via Exo can modulate apoptosis in immune cells. SARS-CoV-2 infected cells can shed Exo with certain viral RNA and high content of Orf2, 3a, 4, 5, 6, 7a, and 9 proteins and ACE2^+^ [229, 230]. It has been shown that SARS-CoV-2 Orf6 can neutralize the nuclear translocation of STAT1 and 2 and suppress the expression of IFN-stimulated genes, leading to impaired cellular immunity against viruses [231]. The transfer of viral particles inside circulating Exo is related to the lack of direct physical interaction of viral antigens with antibody-producing lymphocytes as seen in COVID-19 patients. It is noteworthy to mention that most contents of Exo RNAs remain intact during viral infection, indicating that the immune-modulatory properties of Exo are still stable [232]. As such, it can be concluded that the exosomal delivery of viruses can contribute to the distribution of infectious particles using either viral or Exo equipment.

In addition to supporting the role of Exo in the propagation of viral infections, virus-carrying Exo can also act as a decoy for immune cells. For example, the production of Exo with a specific proteomic signature containing high levels of ACE2, also known as defensosomes, has been indicated. The Exo can prohibit the entry of SARS-CoV-2 to other cells via direct binding spike protein on the virus surface [230]. Besides, recently it was suggested that circulatory Exo isolated from COVID-19 patients contained higher proteins associated with inflammation, coagulation, and immune response [229]. To this end, Barberis and co-workers found a significant increase in levels of certain factors such as alpha-1-acid glycoprotein 1, C-reactive protein, lysozyme C, titin, and zinc-alpha-2-glycoprotein in Exo isolated from COVID-19 patients. They also showed the increase of IL-12, NO, ROS, and elevation of factors associated with coagulation, prothrombin activation, and clathrin-mediated endocytosis [229]. Unlike non-immune cells, effector APCs can introduce viral epitopes inside EVs during antigen processing. EVs containing viral epitopes can be easily reached by neighboring APCs at proximity or remote sites and frustrate them in the absence of complete virion or necessary elements, resulting in provoking acquired immunity (Fig. 8). Along with these statements, the entry of APC-associated EVs into the uninfected non-APCs can trigger specific signaling pathways that limit viral replication and transmission [233]. Exo-carrying viral genetic materials such as miRNAs, lncRNAs, and circular RNAs can induce anti-viral immunity inside the target cells [234]. The elevated miR-423-5p contents in Exo-isolated from rabies virus infection can limit viral infection via the induction of the interferon signaling pathway [235]. In an experiment, the incubation of human macrophages with IFN-α produces Exo that are rich in DNA cytidine deaminase APOBEC3G, leading to the protection of hepatocytes against HBV [236]. The existence of receptors on the Exo surface can also limit virus infection. For example, it was shown that T cell-derived Exo can carry CD4 with the potential to bind and neutralize HIV virus. On the other hand, the infection of host cells with HIV and production viral Nef can per se reduce the levels of CD4 on Exo surface and promote viral infectivity [237].

## Conclusion

Despite recent advances in the understanding of the intersection between viral replication and Exo biogenesis, the exact underlying mechanisms remain to be understood. It seems that Exo can act as natural bioshuttles to cover the viral particles in direct contact with immune cells and increase the entry of viruses due to surface ligands which are more important for envelope viruses. On the other hand, the existence of other signaling molecules can limit the virus’s propagation to non-infected cells. These features show the opposing effects of Exo on viral infections and more investigations are highly recommended to address these issues.

## Data Availability

All data generated or analyzed in the manuscript are included in this article.

## Abbreviations

AP-2: Adaptor protein-2
AAV2: Adeno-associated virus-2
ACE2: Angiotensin-converting enzyme 2
AWOL: Autophagosome-mediated exit without lysis
CVB3: Coxsackievirus B3
DPP4: Dipeptidyl peptidase 4
dsDNA: Double-stranded DNA
ESCRT: Endosomal sorting complexes required for transport machinery
EV71: Enterovirus 71
EBV: Epstein–Barr virus
Exo: Exosomes
ECM: Extracellular matrix
EV: Extracellular vesicles
FAK: Focal adhesion kinase
GPCRs: G-protein-coupled receptors
HSC70: Head shock cognate protein 70
HSPs: Heat shock proteins
HAV: Hepatitis A virus
HCV: Hepatitis C Virus
HEV: Hepatitis E virus
HRS: HGF-regulated tyrosine kinase substrate
HD-PTP: Histidine domain protein tyrosine phosphatase
HHV-6: Human herpes virus
ILVs: Intraluminal vesicles
MVs: Microvesicles
mtDNA: Mitochondrial DNA
MVBs: Multivesicular bodies
nSMase 2: Neutral sphingomyelinase 2
PI3K: Phosphatidylinositol 3-kinase
PI3P: Phosphatidylinositol-3-phosphate
PKC: Protein kinase C
eHAV: Quasi–enveloped virion
Rob1: Receptor tyrosine kinase-like orphan receptor 1
RTKs: Receptors of tyrosine kinases
STAM: Signal transducing adaptor molecule
SV40: Simian virus 40
ssDNA: Single-stranded DNA
SNAREs: Soluble N-ethylmaleimide-sensitive factor attachment protein receptors
TEM: Tetraspanin membrane microdomains
TMPRSS2: Transmembrane protease serine subtype 2
VAMP: Vesicle-associated membrane proteins
